# Flash-Induced High-Throughput Porous Graphene via Synergistic Photo-Effects for Electromagnetic Interference Shielding

**DOI:** 10.1007/s40820-023-01157-8

**Published:** 2023-08-02

**Authors:** Jin Soo Lee, Jeong-Wook Kim, Jae Hee Lee, Yong Koo Son, Young Bin Kim, Kyoohee Woo, Chanhee Lee, Il-Doo Kim, Jae Young Seok, Jong Won Yu, Jung Hwan Park, Keon Jae Lee

**Affiliations:** 1 Department of Materials Science and Engineering, Korea Advanced Institute of Science and Technology (KAIST), 291 Daehak-ro, Yuseong-gu, Daejeon, 34141 Republic of Korea; 2 School of Electrical Engineering, Korea Advanced Institute of Science and Technology (KAIST), 291 Daehak-ro, Yuseong-gu, Daejeon, 34141 Republic of Korea; 3 Department of Printed Electronics, Nano-Convergence Manufacturing Systems Research Division, Korea Institute of Machinery and Materials (KIMM), 156 Gajeongbuk-Ro, Yuseong-Gu, Daejeon, 34103 Republic of Korea; 4 Department of Mechanical System Design Engineering, Seoul National University of Science and Technology, 232 Gongneung-ro, Nowon-gu, Seoul, 01811 Republic of Korea; 5 Department of Mechanical Engineering (Department of Aeronautics, Mechanical and Electronic Convergence Engineering), Kumoh National Institute of Technology, 61 Daehak-ro, Gumi, Gyeongbuk 39177 Republic of Korea

**Keywords:** Porous graphene, Flash lamp, Photo-pyrolysis, High-throughput, Electromagnetic interference shielding

## Abstract

Porous 2D materials with high conductivity and large surface area have been proposed for potential electromagnetic interference (EMI) shielding materials in future mobility and wearable applications to prevent signal noise, transmission inaccuracy, system malfunction, and health hazards. Here, we report on the synthesis of lightweight and flexible flash-induced porous graphene (FPG) with excellent EMI shielding performance. The broad spectrum of pulsed flashlight induces photo-chemical and photo-thermal reactions in polyimide films, forming 5 × 10 cm^2^-size porous graphene with a hollow pillar structure in a few milliseconds. The resulting material demonstrated low density (0.0354 g cm^−3^) and outstanding absolute EMI shielding effectiveness of 1.12 × 10^5^ dB cm^2^ g^−1^. The FPG was characterized via thorough material analyses, and its mechanical durability and flexibility were confirmed by a bending cycle test. Finally, the FPG was utilized in drone and wearable applications, showing effective EMI shielding performance for internal/external EMI in a drone radar system and reducing the specific absorption rate in the human body.

**Supplementary Information:**

The online version contains supplementary material available at 10.1007/s40820-023-01157-8.

## Highlights

- Flash-induced porous graphene (FPG) was synthesized via a broad-spectrum flash lamp that induced synergistic photo-effects between ultraviolet and visible-near-infrared wavelengths, resulting in large-area synthesis in just a few milliseconds.
- A hollow pillar graphene with low sheet resistance of 18 Ω sq^−1^ was produced, exhibiting low density (0.0354 g cm^−3^) and outstanding absolute electromagnetic interference shielding effectiveness of 1.12 × 10^5^ dB cm^2^ g^−1^.
- A lightweight, flexible, and high-throughput FPG is applied for electromagnetic interference shielding of a drone radar system and the human body.

**Supplementary Information:**

The online version contains supplementary material available at 10.1007/s40820-023-01157-8.

## Graphical abstract

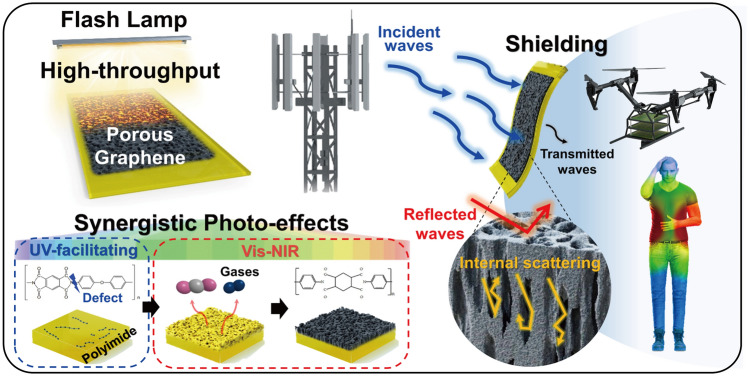

## Introduction

With the advent of future mobility and wearable electronics, various electronic components including sensors, actuators, processors, and controllers are integrated with cloud computing, big data, and 5G wireless communication for autonomous driving, intelligent robotics, and real-time healthcare [1–3]. The highly-integrated multifunctional electronics generate a tremendous amount of electromagnetic interference (EMI), causing critical signal noise, inaccuracy of data transmission, malfunction/failure of the system [4], and health hazards [5–7]. Bulk metal materials have been conventionally used to shield EMI because of their high reflectivity to EM waves. However, the considerable weight and low flexibility of metals restrict the practical demonstration of mobility, aerospace, and wearable applications [8].

A variety of composites have been developed for soft and lightweight EMI shielding materials by incorporating conductive fillers such as metal nanomaterials [9, 10], MXene [11, 12], and graphene [13–15] into the dielectric polymer matrix. The conducting elements of the composite materials enable absorption or reflection of EM waves to shield EMI, while a low-density and flexible polymer constitutes the overall framework. However, the low percolation networks formed between nanoparticles (or 1D wires) and 2D nanosheets with poor dispersion properties lead to low EMI shielding effectiveness (SE). In this regard, porous 2D materials such as MXene foam [16] and porous graphene [17–19] have been proposed for potential EMI shielding materials because the highly conductive pathways and pores existing throughout the materials facilitate effective reflection and internal scattering/absorption of EM waves, respectively.

Recently, light-material interactions have attracted a great deal of attention because they can activate transient, multi-physical, and spatiotemporally controlled reactions [20–23] to structure porous 2D materials with large surface-to-volume ratios [24, 25]. Several research groups have reported that lasers can induce photo-pyrolysis or photo-reduction in precursor materials such as polyimide (PI) [26, 27], phenolic resins [28], lignin [29], and graphene oxide (GO) [30, 31], resulting in the facile formation of porous graphene owing to a rapid temperature rise and local heating. Nevertheless, the laser process is not suitable for low-cost, large-area mass production due to its small spot beam size and time-consuming serial process. From the perspective of scalable light sources, xenon flash lamps are considered promising because of their instantaneous and large-scale processability, as well as compatibility with a roll-to-roll process [32]. In addition, the broad spectrum of the flash lamp has considerable advantages in promoting synergistic photo-thermo-chemical interactions compared to a monochromatic laser [33], showing substantial potential for constructing porous 2D materials in a high-throughput manner regardless of the materials’ optical properties including absorbance and transmittance.

Herein, we report a flash-induced porous graphene (FPG) synthesis method to achieve a high-throughput, outstanding performance, lightweight, and flexible EMI shielding film for drones and wearable applications. The ultraviolet (UV) range of the broad spectrum flashlight led to the breaking of chemical bonds in polyimide (PI) molecules which subsequently created defects for effective light absorption in the visible-near-infrared (Vis–NIR) wavelengths. The photothermal heat generated by the Vis–NIR region caused graphene synthesis while CO_2,_ N_2_ and CO gases were released from the PI film surface, resulting in large area (5 × 10 cm^2^) hollow pillared graphene with a low density of 0.0354 g cm^−3^ in a few milliseconds (ms). The synthesized FPG showed outstanding absolute EMI SE of 1.12 × 10^5^ dB cm^2^ g^−1^ due to its low sheet resistance (18 Ω sq^−1^) and multiple internal scattering. To confirm the FPG formation mechanism, the chemical/structural changes were thoroughly analyzed by using various characterization methods, such as scanning electron microscopy (SEM), X-ray photoelectron spectroscopy (XPS), Fourier transform infrared spectroscopy (FTIR), Raman spectroscopy, transmission electron microscopy (TEM) and X-ray diffraction (XRD). The feasibility of the FPG growth was verified by theoretically calculating the heat distributions of the PI film, which exhibited sufficient surface temperature (up to 2300 ℃) for graphene synthesis. The synthesized FPG was applied for aerospace and wearable applications, including drones and the human body. In a drone radar system, the lightweight FPG film successfully replaced conventional heavy metal jigs by shielding EMI from multi-stacked printed circuit boards (PCBs) and external noise signals. For wearable applications, the FPG effectively reduced the specific absorption rate (SAR) of the human body by 80.3% per 1 g of tissue and also lowered the magnitude of EM waves penetrating the human body by ~ 9.88 dB.

## Experimental Section

### Preparation of Flash-Induced Porous Graphene (FPG)

Commercial polyimide films (PI; Kapton HN500, Dupont) were used for the experiments. PI films were cleaned using ethanol and de-ionized water in an ultrasonic bath. Photo-pyrolysis of the PI film was performed using a flash lamp (Pulseforge 1300, Novacentrix) under ambient conditions. The wavelengths of the flash lamp range from 250 to 1100 nm (ultraviolet to near-infrared; UV to NIR) and the wavelengths are tuned using an optical pass filter (UV-cut window, Novacentrix). The lamp power and pulse width were adjusted in the ranges of 1–16 W cm^−2^ and 1–10 ms, respectively. The fluences were measured using an energy meter for different lamp power and pulse widths. The single irradiation area was set at 5 cm × 10 cm with a square mask for edge clearance. The stage was moved along the Y-axis to obtain a large area FPG of 10 cm × 10 cm. The lamp power, pulse width, irradiation frequency, and stage moving speed were adjusted to be 9 W cm^−2^, 4 ms, 1 Hz, and 100 mm s^−1^, respectively.

### Characterization of FPG

The temperature distribution of PI during flash lamp irradiation was simulated by the finite element method using the heat transfer module of COMSOL Multiphysics 6.0, governed by the following heat transfer Eq. (1) [34–36]:

1 $$ {\rho C}\frac{\partial T}{{\partial t}} - \nabla \cdot\left( {{\text{k}}\nabla {\text{T}}} \right) = Q $$

where ${\uprho }$ is the density, C is the heat capacity, T is the temperature, t is the time, ${\text{k}}$ is the thermal conductivity and Q is the heat flux. The values of temperature-dependent parameters (${\uprho },{\text{ C}},{\text{k}}$) of specific materials (PI, Fe, O_2_) used in the simulation were given by the COMSOL material library. The heat generation per volume from absorbed light was calculated by Eq. (2) [37]:

2 $$ Q\left( {x,y} \right) = \left( {1 - R} \right)I_{0} \alpha_{C} e^{{ - \alpha_{c} y}} $$

where $R$ is the reflectivity, $I_{0}$ is the irradiated lamp intensity, and $\alpha_{C} { }$ is the absorption coefficient. A UV–Visible spectrometer (UV–Vis spectrometer; Lambda 1050, Perkin Elmer) was used to characterize the values for the transmittance, reflectivity, and absorption coefficient of PI. The surface and cross-sectional structures of the samples were observed using field-emission scanning electron microscopy (SEM; S-4800, Hitachi), and the thicknesses were measured by cross-sectional SEM images. High-resolution transmission electron microscopy (HRTEM; Jelec-ARM200F, JEOL) was used to investigate the nano-structures at an accelerating voltage of 200 kV. The Raman spectra were obtained using a Raman spectrometer (LabRAM HR Evolution Vis–NIR, HORIBA) with a 514 nm laser excitation. The surface elemental composition and the chemical state of the samples were examined using X-ray photoelectron spectroscopy (XPS; Axis-Supra, Kratos). A Fourier transform infrared spectroscopy (FTIR; Nicolet iS50, Thermo Fisher Scientific Instrument) was used to collect the FTIR spectra between 800 and 3500 cm^−1^. X-ray diffraction (XRD) analysis was conducted using a RIGAKU Ultima IV with Cu Kɑ radiation (λ = 1.54 Å). The sheet resistance was characterized using a four-point probe (CMT-SR1000N, AIT). A vector network analyzer (VNA; N5222B, Keysight) and waveguides were used to measure scattering parameters (S11 and S21) of the material in the 18–26.5 GHz (K-band) frequency range at room temperature. The EMI SE value was calculated using the given equations [38–40]:

3 $$ {\text{T}} = \left| {{\text{S}}_{21} } \right|^{2} $$

4 $$ {\text{R}} = \left| {S_{11} } \right|^{2} $$

5 $$ {\text{A}} = 1 - {\text{T}} - {\text{R}} $$

6 $$ {\text{SE}}_{R} \left( {{\text{dB}}} \right) = - 10{\text{log}}\left( {1 - {\text{R}}} \right) $$

7 $$ {\text{SE}}_{A} \left( {{\text{dB}}} \right) = - 10{\text{log}}\left[ {{\text{T}}/\left( {1 - {\text{R}}} \right)} \right] $$

8 $$ {\text{SE}}_{T} \left( {{\text{dB}}} \right) = {\text{SE}}_{R} + {\text{SE}}_{A} $$

9 $$ {\text{A}}_{{{\text{eff}}}} = \left( {1 - {\text{R}} - {\text{A}}} \right)/\left( {1 - {\text{R}}} \right) $$

T, R, and A represent the transmission, reflection, and absorption coefficients, respectively. SE_R_, SE_A,_ and SE_T_ are reflection, absorption, and total EMI SE, respectively. The effective absorbance (A_eff_) is calculated to evaluate the absorption potential of materials. The home-built moving stage was used for bending the FPG.

### Preparation of EMI Shielding Properties for Drone Radar and Human Body

A K-band frequency-modulated continuous wave (FMCW) radar on a drone (Pixhwak) operating from 23 to 25 GHz was used to verify the EMI shielding properties for a machine [41]. As shown in Fig. S1, the radar PCB consisted of a power board, a power amplifier board, a radio frequency (RF) transceiver board, and antennas. The power amplifier board was composed of a power amplifier (HMC943ALP5DE) and a bias controller (HMC980). The RF transceiver board was divided into transmitter parts and receiver parts. The transmitter was implemented with a crystal clock (ECS-TXO-5032MV), a microcontroller unit (MCU, STM32F215RGT6), a frequency synthesizer (ADF4158), and a voltage-controlled oscillator (HMC739LP4). Through the Wilkinson divider, the transmitted signal is divided into a local oscillator and a power amplifier. The receiver was fabricated with an I-Q down converter (HMC977LP4E) and a bandpass filter. The bandpass filter was designed using a 2-stage operational amplifier (OP Amp, LTC6226) that operates from 5 kHz to 2 MHz. Universal software radio peripheral (USRP) N210 was employed for an analog-to-digital converter (ADC) and field-programmable gate array (FPGA). The number of bits of the ADC was 14 and the sampling frequency was 5 MHz. The 3-dimensional dielectric resonator antenna (DRA) was designed to achieve low feeding network loss, low weight, and high gain.

The internal EMI from the RF transceiver board was observed using an open-ended probe antenna (OEW-42). The 2-dimensional electric fields radiated from the RF transceiver PCB were measured in a 20 × 20 cm^2^ area by controlling the position of the probe antenna, as shown in Fig. S2 [42, 43]. The antenna gains of standard horn antennas without the FPG and with the FPG were measured to test the shielding performance of external EMI entering the antenna. The various types of standard horn antennas (Narda and MTG for 5.8–40 GHz) and open-ended probe antennas (OEW for 5.8–40 GHz) were used, as the detailed frequency range of each antenna is shown in Fig. S3. To evaluate the external EMI shielding performance for a drone radar system, the experiment setup was implemented as shown in Fig. S4. A power divider was used to divide the transmitting signal and external EMI signal. A waveguide antenna was employed to transmit the external EMI signal to the receiver antenna of the radar. The FPG was applied between the waveguide antenna and the receiver antenna of the radar.

To verify the EMI shielding performance for a human body, the SAR was measured [44]. As shown in Fig. S5, the signal source was implemented as a signal generator (N5171B) and a power detector (U8481A) was employed as the signal receiver. For the transmitting antenna, a dipole antenna (D5GHZV2) was adopted, and for the receiving antenna, a probe antenna (EX3DV4) was selected. A robot arm to control the position of the probe antenna was implemented by TX90XL and for a phantom, which is a solution with similar properties to human tissue, ELI Phantom V6.0 was employed. In addition, to measure the magnitude of an EM wave transmitted through a human body, a horn antenna (HF907) and a vector network analyzer (R&S ZNA43) were used.

## Results and Discussion

### FPG Fabrication Mechanism by Synergistic Photo-Effects

**Fig. 1:**
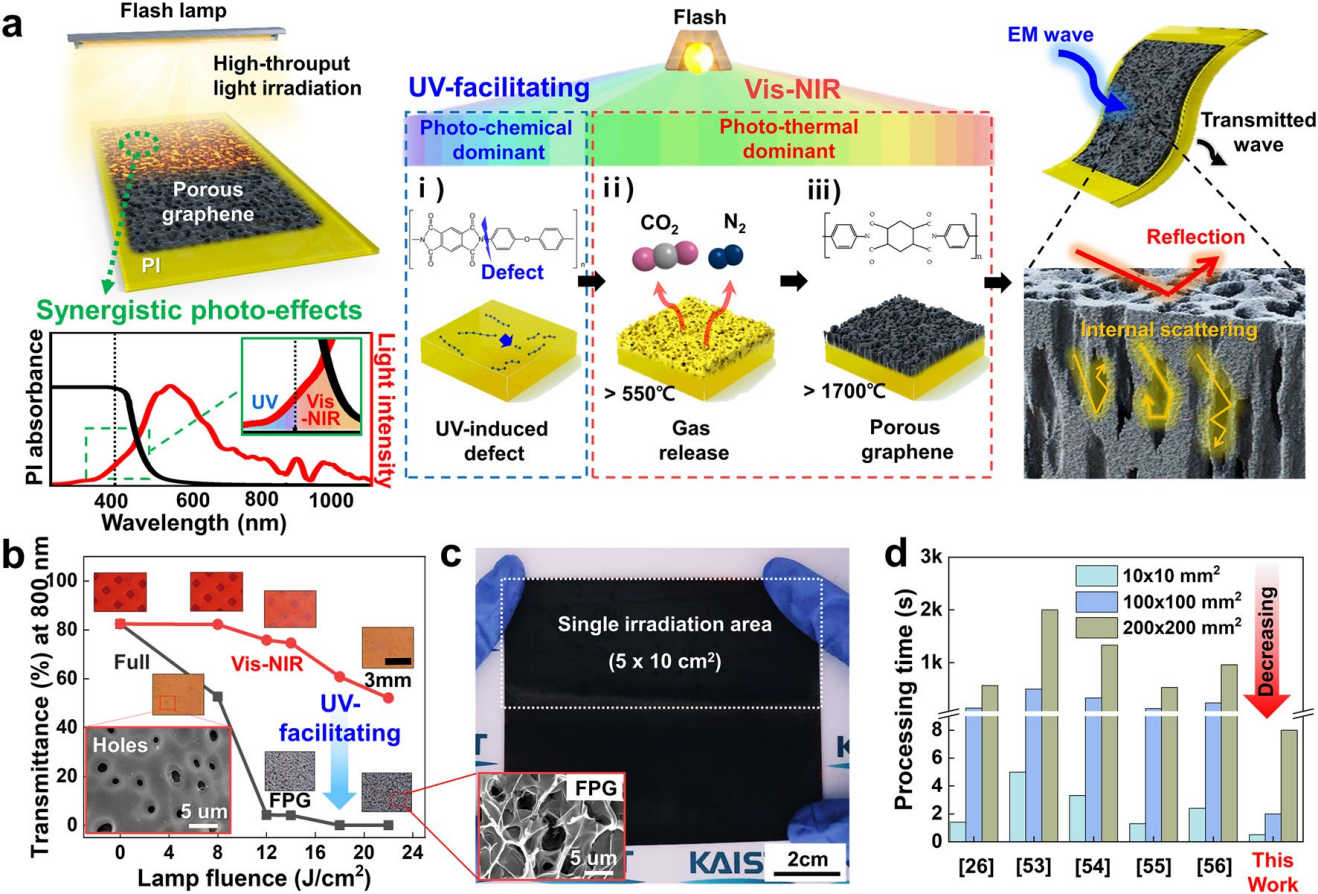
Overall concept of FPG fabrication. **a** Schematic of the photo-pyrolysis process, facilitated by synergistic photo-effects and its application. The fabrication process can be broadly divided into three steps: (i) UV-induced photochemical reactions, (ii) Vis–NIR-induced photothermal reactions, and (iii) porous graphene synthesis. **b** Optical transmittance (at 800 nm wavelength) of PI irradiated under various lamp fluences from 0 to 22 J cm^−2^ using Vis–NIR and full spectrum. The inset photos show the PI film after irradiation by flash lamp. The inset SEM image presents the generation of holes. **c** Photo of the FPG with a size of 10 × 10 cm^2^, fabricated using a single irradiation area of 5 × 10 cm^2^. The inset shows the surface morphology of the FPG. **d** Comparison of processing time with calculation between this work and previous reports using a laser [26, 53–56]

Figure 1a schematically illustrates the overall concept of the FPG synthesis inducing synergistic photo-effects to the PI film for the fabrication of EMI shielding materials. Broad-spectrum flash irradiation generates sequential photoreactions into the PI film through the dual absorption of UV and Vis–NIR, resulting in the synthesis of porous graphene. Depending on the flash spectrum region, three different photo-effects are induced as follows: (i) UV-induced photochemical reactions, (ii) Vis–NIR-induced photothermal reactions, and (iii) porous graphene synthesis. At the beginning of flash exposure, photochemical and photothermal reactions occur, with the dominant reaction determined by the light energy and wavelength [45]. At low energy, UV wavelengths (over 3.5 eV) break down the low-bond energy C–N (3.04 eV) [46], leading to the formation of micro-cracks and changes in surface morphology [47, 48]. These physical and chemical changes affect the optical properties of the PI film, increasing its absorbance at Vis–NIR wavelengths [49]. Therefore, as the light energy increases, Vis–NIR wavelengths generate subsequent or synergistic photothermal reactions by accumulating heat in the PI film [50–53]. The absorbed light energy gradually increases heat accumulation, and when the PI film reaches 550 °C, CO_2_, N_2_, and CO gases are released by dissociation of C=O, C–N, and C–O bonds [54, 55]. Carbonization begins at temperatures above 800 °C, involving the breakdown of complex PI chains into simpler carbon structures. This process expels volatile components and leaves disordered, carbon-rich residue. When the temperature exceeds 1700 °C [56–58], the active emission of gases forms a porous structure, and the amorphous carbon (*sp*^3^) transforms into stacked, ordered graphene sheets (*sp*^2^). This process results in the formation of porous graphene, a crystalline structure of carbon atoms partially arranged in a hexagonal lattice [59]. The synthesized porous graphene can be applied to EMI shielding by reflecting and scattering incident EM waves at its external and internal surfaces.

To confirm the synergistic photo-effects, the full wavelength, and the Vis–NIR wavelength by blocking UV of the flash lamp were individually irradiated on the PI film with lamp fluence of pristine, 8, 12, 14, 18, and 22 J cm^−2^. As displayed in Figs. 1b and S6, a significant reduction in the transmittance of the PI film was observed after the full wavelength of flash irradiation, compared to the Vis–NIR wavelength of irradiation. This indicates that the UV spectral region facilitated 800 nm wavelength flash absorption into the PI film. As shown in the inset optical and SEM images of Fig. 1b, the color of the PI film was changed from red, and orange to gray with increasing flash fluence due to the sequential formation of holes and porous graphene [60]. Note that porous graphene was only formed on the PI film with the full wavelength of flash irradiation, suggesting a UV-accelerated photo-pyrolysis effect.

To achieve effective EMI shielding in various applications such as future mobility and wearable devices, a large-area EMI film should be fabricated with high productivity. Figure 1c shows a 10 × 10 cm^2^ FPG manufactured by irradiation of a flash twice with a beam area of 5 × 10 cm^2^. The SEM image in the inset of Fig. 1c demonstrates well-formed graphene with a porous structure in the flash irradiation area. PI film, with its high thermal and chemical stability, is suitable to implement lightweight and flexible EMI shielding materials. Its high characteristics minimize the influence of environmental conditions during the preparation process, thus it is widely used in various electronic device applications [61]. The PI film is compatible with mass production processes, including roll-to-roll, facilitating easy acquisition of commercialized film types of varying thicknesses and compositions. The chemical structure of PI is particularly suitable for generating porous graphene through photo-pyrolysis due to the presence of aromatic *sp*^2^ carbons. These are more likely to form the hexagonal graphene structure than other precursors, which primarily generate amorphous carbon.

Due to its high thermal stability, PI is not greatly affected by temperature conditions. However, if the temperature is artificially raised to preheat the PI, it could potentially enhance the speed of the photo-reaction, which could in turn affect the rate of gas emission and the pore expansion time [62]. The flash lamp's instantaneous high-temperature increase can effectively remove the moisture absorbed in the polymer, thus reducing the impact of humidity. However, long-term exposure in high humidity could induce hydrolysis of the PI, leading to increased light transmittance and potentially reducing light absorption [63]. In the process of photo pyrolysis reactions, the presence of oxygen enhances the oxidation reaction, thereby generating more heat. It has been reported that the resultant heat increase from oxidation enlarges the pore size and facilitates deeper heat transfer [64].

Figure 1d shows the comparison of the processing time between previously reported laser fabrications [26, 65–68] and this work for the synthesis of porous graphene with dimensions of 10 × 10, 100 × 100, and 200 × 200 mm^2^. The difference in production time was negligible in the small area of 10 × 10 mm^2^, while a significant decrease of more than 66.5 times was confirmed in large areas of 100 × 100 and 200 × 200 mm^2^. Figure S7 shows the calculation method and Table S1 presents the detailed calculation results. Note that the flash can dramatically increase productivity by increasing the size of the lamps and chamber, or by application to roll-to-roll processes. Table S2 shows comparison of porous graphene formation method of this work with previously reported light-induced method. These results suggest that the flash lamp is a suitable light source to fabricate a porous graphene-based EMI film with a large area and high productivity.

### Characterization of the FPG

**Fig. 2:**
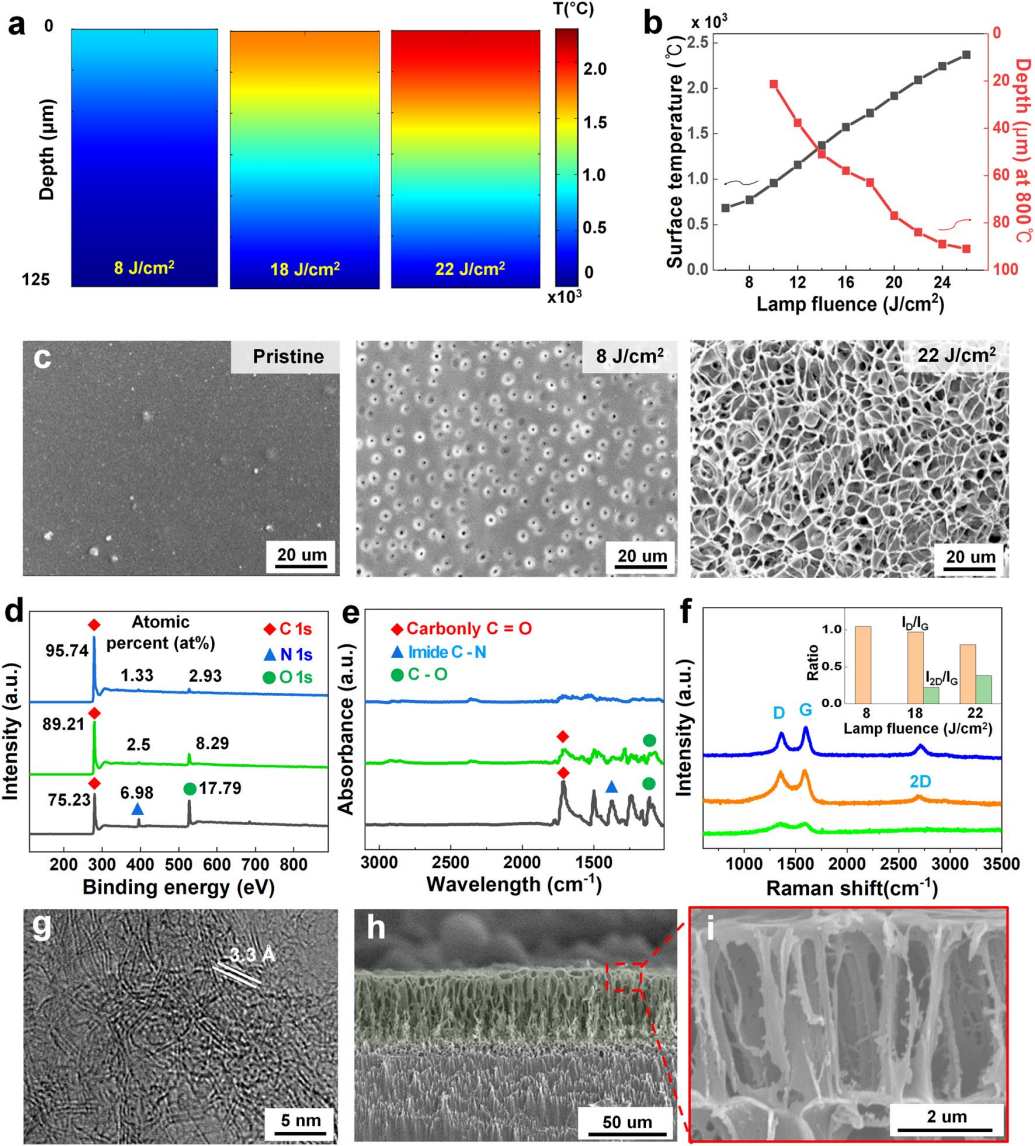
Characterization of the FPG. **a** Simulation results of temperature distribution in PI under fluence of 8, 18, and 22 J cm^−2^. **b** Surface temperature and depth of carbonization by COMSOL simulation for each lamp fluence. **c** Top-view SEM images of PI irradiated at a fluence of pristine, 8, and 22 J cm^−2^. **d** XPS spectra with atomic percent (at%), **e** FTIR spectra obtained at a fluence of pristine (black line), 8 (green line), and 22 (blue line) J cm^−2^. **f** Raman spectra of the FPG at a fluence of 8 (green line), 18 (orange line), and 22 (blue line) J cm^−2^. Inset shows the *I*_D_/*I*_G_ and *I*_2D_/*I*_G_ ratio at each lamp fluence. **g** Top-view HRTEM images of FPG with domain spacing of 3.3 Å at a lamp fluence of 22 J cm^−2^. **h** Cross-sectional SEM image reveals a ~ 62 μm thick hollow pillar FPG at a lamp fluence of 22 J cm^−2^. **i** Magnified SEM image showing the porous hollow pillar morphology including ~ 5 µm pores in the FPG

To confirm whether a temperature of 1700 °C, necessary for graphene formation, can be achieved using a lamp, we conducted a COMSOL simulation to evaluate the maximum temperature and temperature distribution at various depths. Theoretical calculations were performed to determine the thermal distribution within a 125 µm-thick PI under various flash fluences ranging from 6 to 26 J cm^−2^. As depicted in Fig. 2a, a gradual increase in temperature distribution with depth was observed as the lamp fluence increased. As illustrated in Fig. 2b, the maximum surface temperature reached 2300 °C at a lamp fluence of 26 J cm^−2^, with heat transfer occurring at roughly 100 µm depth within the PI. These results indicate that the necessary temperature for graphene formation can be achieved using lamp irradiation. Figure 2c presents top-view SEM images of the PI film after flash irradiation under various lamp fluences of pristine, 8 and 22 J cm^−2^. As the lamp fluence increased, ~ 5 µm-sized micro-pores began to form and were finally synthesized into porous structural walls that were hundreds of nanometers wide. To further characterize the porous structure, we verified the pore size by lamp fluence, as shown in Fig. S8. As the lamp fluence increased from 18 to 24 J cm^−2^, the pore size increased from approximately 1.4 to 19.2 µm.

The chemical changes and structural characteristics were analyzed via XPS, FTIR, Raman spectrum, TEM and XRD to demonstrate the synthesis mechanism of micro-holes and porous materials. Figure 2d shows the XPS results with major peaks at a binding energy of 285 eV for C 1*s*, 397 eV for N 1*s*, and 532.5 eV for O 1*s*, which correspond to the main elements of carbon, nitrogen, and oxygen on the surface of the FPG [69]. As the fluence increased, the atomic percent of C rose from 75.23 to 95.74 at%, while the atomic percent of N and O decreased from 6.98 to 1.33 at% and from 17.79 to 2.93 at%, respectively. Low oxygen content signified a difference from graphite oxide, which possesses functional groups [70]. Results from elemental mapping using SEM–EDS also showed a mass percentage of carbon of 96.2%, as demonstrated in Fig. S9. Figure 2e shows the FTIR results with specific peaks ranging from 1090 to 1776 cm^−1^ indicating the stretching and bending modes of C–O, C–N, and C=O bonds. After lamp irradiation, a broad absorption spectrum ranging from 1000 to 1700 cm^−1^ was observed. At a fluence of 22 J cm^−2^, the peak associated with C=O, C–O, and C–N noticeably decreases. The XPS and FTIR results demonstrate that the carbon content becomes dominant due to the gas release accompanied by a decrease in nitrogen and oxygen content [71].

Figure 2f shows the results of Raman spectra with three major peaks of the graphitic material: the D peak caused by defects, the primary in-plane vibrational G peak, and a two-phonon scattered 2D peak, located at around 1360, 1581, and 2675 cm^−1^, respectively [27]. In particular, the 2D peak can indicate the formation of graphene, not amorphous carbon or graphite oxide [72–74]. As the fluence increased, the D and G peaks appeared, and at fluences of 18 and 22 J cm^−2^, the 2D peak was observed. The inset graph shows the ratios of *I*_D_/*I*_G_ and *I*_2D_/*I*_G_ Raman peaks, which indicate defects in graphene materials and the number of layers in graphene [75, 76]. As the fluence increased, the ratio of *I*_D_/*I*_G_ decreases from 1 to 0.8, and the ratio of *I*_2D_/*I*_G_ increased from 0 to 0.38. The *I*_2D_/*I*_G_ ratio of 0.38 indicates the formation of 4–5 layers of graphene [72]. Figure 2g displays HRTEM images, revealing the presence of wrinkles across a large area and exhibiting a domain spacing of 3.3 Å. These features—wrinkles and domain spacing—suggest that the FPG is not amorphous carbon [77]. The crystallinity of the material can be confirmed through XRD peaks. Figure S10 shows an intense peak centered at 2θ = 26°, giving an interlayer spacing (d) of ~ 3.4 Å between (002) planes in the FPG, indicating a high degree of graphitization. The peak at 2θ = 43° is indexed to (100) reflections which are associated with an in-plane structure [78]. The Raman spectra, TEM images and XRD spectra indicate that a multi-layered graphene structure has formed through rearrangement in a disordered carbon matrix [26, 79]. Table S3 provides the comparison of the analysis results of our FPG with those of previous studies on light-induced graphene.

Figure 2h shows cross-sectional SEM images of the FPG obtained at a fluence of 22 J cm^−2^. Vertically aligned porous hollow pillar structures were formed because the pores expanded and grew in the direction perpendicular to the surface where the gas was released. The thickness of the hollow pillar FPG was formed to ~ 62 μm from the 125 μm thick PI film at a fluence of 22 J cm^−2^. Figure S11 shows cross-sectional images for thickness measurement. The SEM image in Fig. 2i is a magnified cross-sectional view of hollow pillars near the surface, revealing pores with ~ 5 μm size. These results suggest that sufficient temperature was achieved for synthesizing graphene via the flash lamp, resulting in the formation of porous graphene through the rearrangement of remaining carbon and the release of gas.

### EMI Shielding Performance of the FPG

**Fig. 3:**
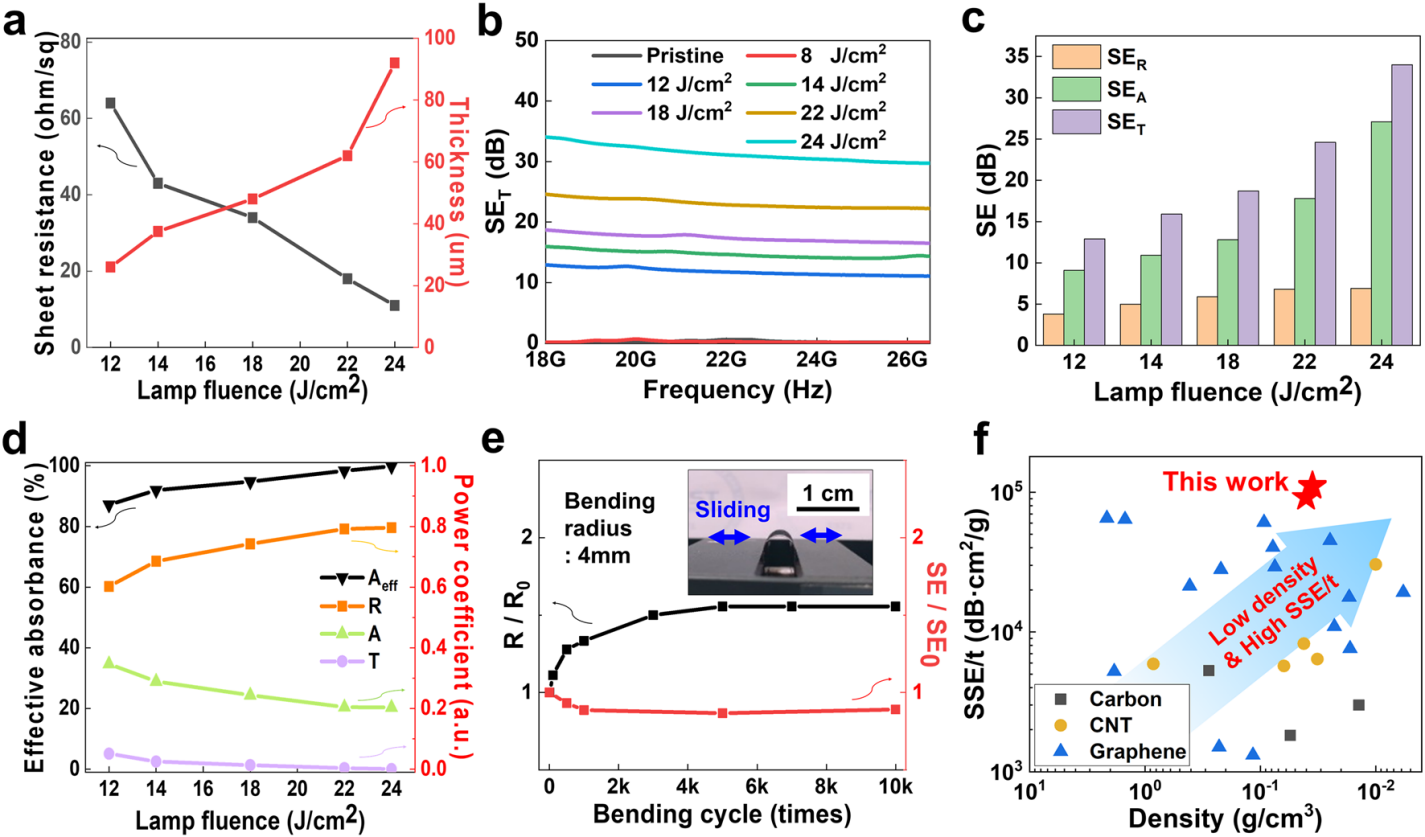
EMI shielding performance of the FPG. **a** The sheet resistance and thickness of the FPG at various lamp fluences of 12, 14, 18, 22, and 24 J cm^−2^. These properties are closely related to the reflection and absorption of EM waves. **b** EMI SE_T_ at K-band under different lamp fluences from 0 to 24 J cm^−2^. A higher SE_T_ value indicates greater EMI shielding performance. **c** Comparison of EMI SE_R_, SE_A_, and SE_T_ at various fluences of 12, 14, 18, 22, and 24 J cm^−2^_._ SE_R_, SE_A,_ and SE_T_ are reflection, absorption, and total EMI SE, respectively. **d** Comparison of R, A and T coefficients and effective absorbance (*A*_eff_) under different fluences of 12, 14, 18, 22, and 24 J cm^−2^. *R*, *A*, and *T* represent the reflection, absorption, and transmission coefficients, respectively. **e** Changes in sheet resistance and SE_T_ during 10,000 cycles of bending. The inset is an optical image of the FPG in a bending state. **f** Comparison of absolute EMI SE (SSE/t) of this work with previously reported carbon-based shielding materials

High EMI shielding properties of porous graphene can be achieved by low sheet resistance and high thickness which enables enhancement of the surface reflection and internal absorption, respectively [80–82]. Figure 3a shows the changes in the sheet resistance and thickness of the FPG at different lamp fluences. As the lamp fluences were increased, the sheet resistance decreased from 64 to 11 Ω sq^−1^ while the thickness increased from 26 to 92 µm. As the lamp fluence increased, the electrical conductivity also increases, as shown in Fig. S12. Based on these results, the EMI SE_T_ of the FPG was evaluated at a frequency range from 18 to 26.5 GHz, the K-band mainly used for 5G/6G communications, as shown in Fig. 3b. As the flash fluence increased, the SE_T_ was continuously enhanced over the entire frequency range. Figure S13 depicts the EMI SE measured in the C-band (4 to 8 GHz), with the measured results confirming SE performance of over 20 dB, comparable to the K-band.

To confirm the dominant factors contributing to the improvement of EMI SE_T_ of the FPG, the EMI shielding performance of SE_R_ and SE_A_ as well as the power coefficient were measured. Figure 3c presents the SE_R_, SE_A_, and SE_T_ of the FPG under various fluences of 12, 14, 18, 22 and 24 J cm^−2^. As the fluence increased, SE_T_ rose from 12.9 to 34 dB, and SE_A_ also elevated from 9.1 to 27.8 dB, while SE_R_ consistently remained below 7 dB. These results confirm that SE_A_ primarily drove the increase in SE_T_. Figures 3d and S14 displays the *A*_eff_ and power coefficients (*R*, *A*, and *T*), which are used to explain the EMI shielding mechanism of the FPG. Throughout the fluence range from 12 to 24 J cm^−2^, the *R* values were always higher than the *A* values. As the fluence increased, *R* values increased gradually while A and *T* values decreased, indicating that surface reflection plays a more significant role than absorption and that lower resistance induced by lamp fluence leads to higher surface reflection. The absorption and reflection are the two main mechanisms influencing EMI shielding. Among these, reflection is enhanced by high electrical conductivity, leading to increased reflection of EM waves and, consequently, a larger *R* coefficient. In shielding materials where reflection dominates [83, 84], if the *R* significantly increases and the *T* decreases, the *A* may decrease according to Eq. (5). However, the SE_A_ value can still increase, as given Eq. (7) [85, 86]. The A_eff_ value, which represents the absorption effectiveness inside the material, excluding surface reflection, increased from 87.5 to 99.8%. This suggests that EM waves are attenuated by internal scattering due to the hollow pillar structure, which increases the propagation path. To verify the EMI shielding characteristics solely due to porosity, we fabricated samples without micro-pores at a similar thickness to those produced with a lamp fluence of 22 J cm^−2^ and compared their EMI shielding characteristics. Figure S15a, b shows surface and cross-sectional SEM images of without micro-pores was fabricated by adjusting the lamp parameters. As shown in Fig. S16a, the sample without micro-pores had a higher R coefficient, and a lower *A* coefficient. The SE_T_ values of the samples, both with and without micro-pores, were similar, but the sample without micro-pores exhibited a lower SE_A_ value, as shown in Fig. S16b. These results indicate that the porous structure influences EM wave absorption. These findings support the shielding mechanism of the FPG [87–89], as shown in Fig. 1a. First, the incident EM wave is reflected from the material surface due to its low resistance. The EM wave is then attenuated at the inside of the material due to the increased path length caused by the high surface area of the porous structure. Finally, the EM wave that is not reflected or attenuated passes through the material, becoming the cause of EM wave interference.

To verify the mechanical durability of the FPG, a bending cycle test was performed at a bending radius of 4 mm, as shown in the inset image of Fig. 3e. After undergoing 10,000 cycles of bending and unbending, with no delamination of the FPG, minor changes in the EMI properties were observed, as indicated by a 1.4 times increase in sheet resistance and a 0.86 times decrease in EMI SE_T_. However, the EMI SEs were higher than the 20 dB, a value that is suitable for practical applications. Figure S17 shows the SE_T_ values as a function of the number of bending cycles. To examine the impact of bending, we bent the FPG at various bending radii, ranging from 5 to 1 mm. Figure S18 shows the changes in sheet resistance and EMI SE after bending. Figure S19a–c are SEM surface images after bending at 5, 3, and 1 mm, respectively. From a bending radius of 5 mm down to 1 mm, the EMI SE decreased due to the formation of cracks when the bending radius was less than 3 mm.

It is important to achieve a high SE under minimal thickness and low density for the effectiveness and applicability of EMI shielding materials to future mobility and wearable electronics [90–92]. The unit of SE alone is not sufficient as it does not consider critical factors such as the material's density and thickness. To address this, a more realistic parameter [93], known as absolute specific SE (SSE/t), is applied to compare the EMI shielding performance among carbon-based EMI shielding materials, as shown in Fig. 3f and Table S4. The unit of SSE/t considers three important parameters—EMI SE, density, and thickness—and is commonly used to evaluate the EMI shielding characteristics of thin and lightweight materials [94–98]. The FPG showed the highest SSE/t value of 1.12 × 10^5^ dB cm^2^ g^−1^, which was calculated by Eq. (10):

10 $$ {\text{SSE}}/{\text{t}} = {\text{SE}}/\left( {{\text{thickness}} \times {\text{density}}} \right) $$

where the density and thickness of the FPG were 0.0354 g cm^−3^ and 62 µm, respectively. The outstanding EMI shielding performance of the FPG can be attributed to the hollow pillar structure enabling multiple internal EM wave scattering and low material density. These results suggest that the lightweight, flexible FPG can be applied to the curvy surfaces of drones and human body for aerospace and wearable applications.

### EMI Shielding Performance for the Drone Radar

**Fig. 4:**
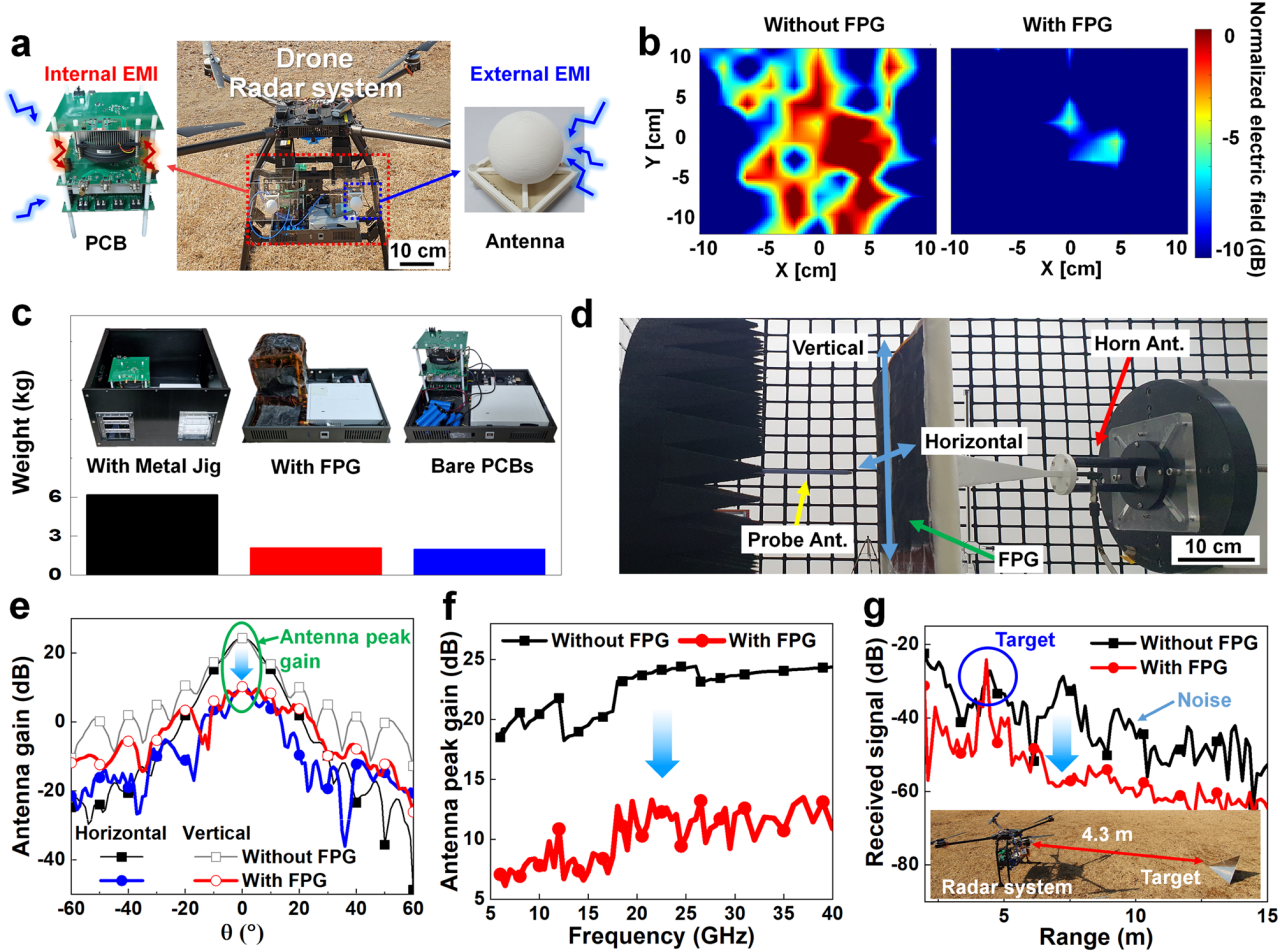
EMI shielding experiment for drone radar. **a** Photos of the K-band drone radar system. The K-band radar consisting of PCB and antenna (Ant.) is exposed to internal/external EMI. **b** Measured 2-dimensional normalized electric field distribution in 20 × 20 cm^2^ of PCB without and with the FPG for the internal EMI shielding experiment. **c** Photos and measured weight of radar system with conventional metal jig, FPG-wrapped PCBs, and bare PCBs. **d** Experimental environment of measuring the standard horn antenna gain. The probe antenna moving in horizontal and vertical directions measured the EM wave from the standard horn antenna with and without the FPG. **e** Measured antenna gain of standard horn antenna at 24 GHz to horizontal angle and vertical angle. **f** Measured antenna peak gain across the frequency range of 5 to 40 GHz. **g** Result of the radar signal received by external EMI and the inset shows a distance of 4.3 m from the target to the drone radar with the intentional external EMI source

In drone radar systems, internal/external EMIs by PCBs and wireless antenna systems cause critical performance degradation by internal circuit oscillation, harmonic noise generation, and heat generation [99, 100]. To evaluate the practical application of the FPG to a drone radar system, the FPG films were integrated into PCBs and antennas, as shown in Fig. 4a. The electric fields radiated from the PCB were measured in a 2-dimensional area of 20 × 20 cm^2^ for the internal EMI shielding experiment, as presented in Figs. 4b and S2. The maximum electric field measured for the FPG-integrated PCB, as shown in Fig. 4b, is reduced by -10.8 dB compared to the bare PCB. Figure 4c displays weights comparisons among conventional metal jig-integrated PCBs, FPG-wrapped PCBs, and bare PCBs. The dimensions of the radar systems incorporating either a metal jig or the FPG are identical, measuring 370 mm × 280 mm × 215 mm. However, the applied FPG is specifically attached to the PCBs, while the conventional metal jig, due to structural limitations, is designed to encase not only the PCBs but the entire radar system as well. The weight of the PCBs wrapped with the FPG was 2.1 kg, comparable to the bare PCBs (2.0 kg) and almost three times lighter than the conventional metal jig-integrated PCBs (6.1 kg). When compared with the same shielding area, the weight of the radar system equipped with the metal jig decreased to 4.14 kg, but it is still heavier than the 2.1 kg radar system using the FPG. When applying a metal thin film for EMI shielding at the same size as the FPG, the weight of the drone radar system was similar, at approximately 2.1 kg. However, due to its excessively high reflectivity, the metal thin film has limitations when applied to communication systems. On the other hand, the FPG, with its absorption characteristics resulting from its porosity, is better suited for application in radar systems. These results indicate that FPG has the potential to replace conventional heavy metal jigs or metal thin film due to its lightweight nature and EMI shielding performance over a large area.

Figure 4d depicts the experimental setup for measuring the antenna gain to evaluate the external EMI shielding performance. The antenna gain refers to the ratio of the radiation intensity in a given angle from the standard horn antenna to the radiation intensity averaged over all angles [101]. The probe antenna moving in horizontal and vertical directions measured the EM wave from the standard horn antenna with and without the FPG. In Fig. 4e, the antenna gains of the standard horn antenna according to the horizontal angle and vertical angle were measured at the main frequency of the radar, 24 GHz. The EMI shielding performance of the FPG reduced the antenna gain at various angles from -60 to 60 degrees, indicating its ability to effectively block EM waves in multiple directions. Figure 4f shows the antenna peak gains across the frequency range of 5 to 40 GHz measured in the experimental setup, as shown in Fig. S3. Frequency bands from 5 to 40 GHz are mainly used for wireless fidelity (Wi-Fi), satellite, long-term evolution (LTE), vehicle-to-everything (V2X), and mmWave 5G communication. The FPG reduced the antenna peak gain across a wide frequency range, particularly showing a reduction of 12.2 dB at the radar main frequency of 24 GHz. The results of the antenna gain measurements suggest that the FPG has the ability to shield EM waves at wide angles and frequencies for antennas in a radar system.

Figure 4g presents the results of the radar signal received by external EMI, and the inset shows the distance of 4.3 m between the target and the drone radar equipped with an intentional external EMI source. In the experimental environment, as shown in Fig. S4, external EM waves from the intentional EMI source caused signal saturation and exceeded the maximum detectable signal power [102–104]. These signal distortions disrupted the distinction between the target signal and noise signals, making it impossible to detect the target from a distance of 4.3 m. To decrease the noise signal of the radar, the FPG was inserted between the intentional external EMI source and the radar receiver antenna, leading to successful target detection. Figure S20 presents an additional experiment applying the FPG to the transmitter of the radar system. These results suggest the possibility of solving both internal and external EMI problems in the aerospace field by applying the large-area, lightweight, and flexible FPG.

### EMI Shielding Performance for the Human Body

**Fig. 5:**
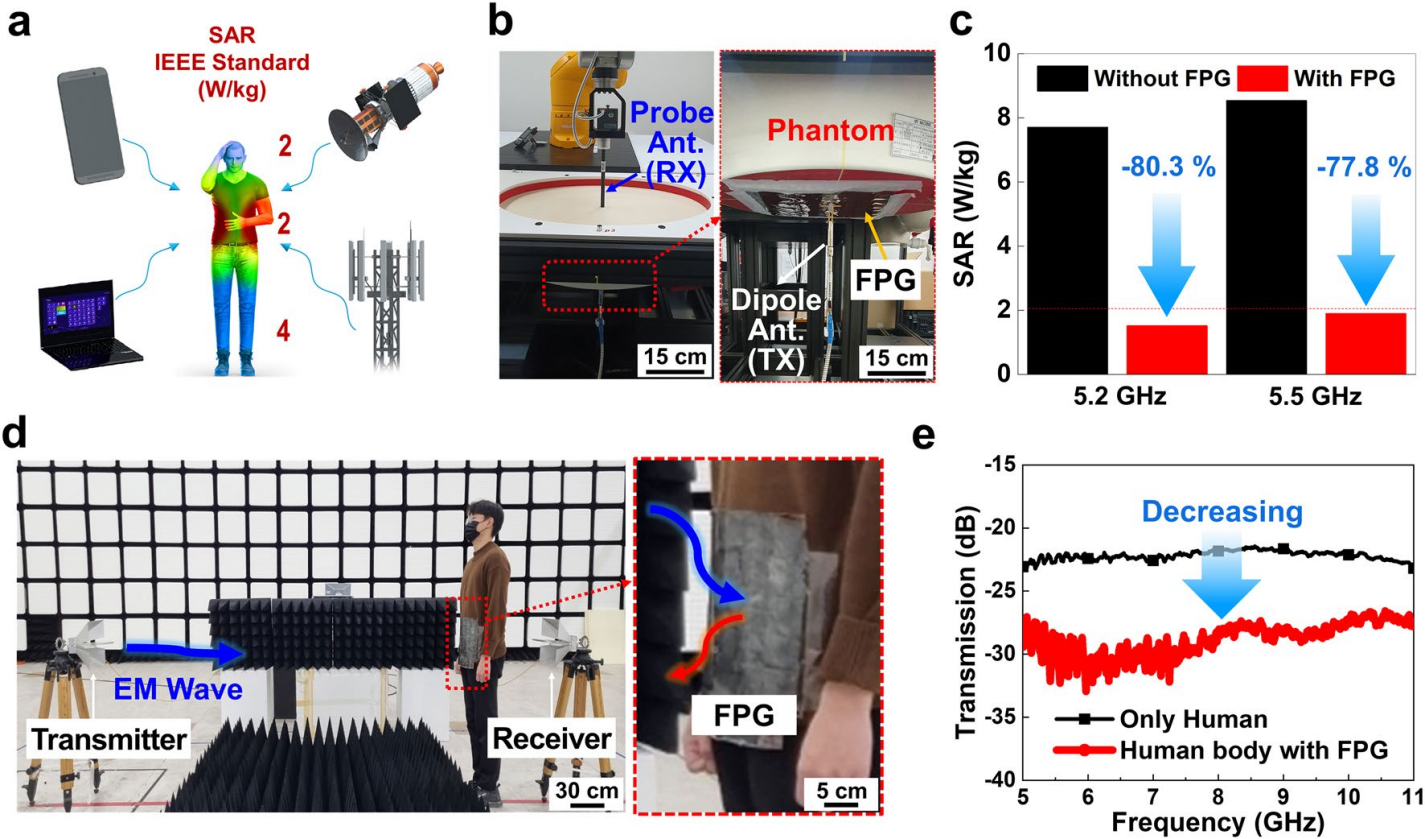
EMI shielding experiment for the human body. **a** Conceptual illustration of the human body affected by EM waves from surrounding electronic devices such as mobile phones, laptop computers, satellites, and base stations. **b** Experimental environment consisting of the dipole antenna, phantom, and probe antenna for SAR measurement. The FPG was placed under the phantom to prevent the propagation of EM signals from the dipole antenna to the probe antenna. **c** Measured SAR at frequencies of 5.2 and 5.5 GHz, which are the representative frequencies for commonly-used indoor and outdoor wireless communication, respectively. **d** Experimental setup for evaluation of the EM wave shielding ability in wearable applications. The FPG was attached to the front of a person's midsection, placed between the transmitter and receiver of EM waves. The FPG could be conformally attached to the human body due to its flexible properties, and its lightweight allowed the person to move freely during the experiment. **e** Measured the transmission from the transmitter antenna to the receiver antenna with and without the FPG in a frequency range from 5 to 11 GHz

Figure 5a is a conceptual illustration of the human body affected by EM waves from surrounding electronic devices such as mobile phones, laptop computers, satellites, and base stations. Since long-term exposure to EM waves can cause skin cancer, cataracts, nervous system disorders, and heart disease, the Institute of Electrical and Electronics Engineers (IEEE) recommends that the SAR (the ratio of energy absorbed per unit mass of biological tissue) does not exceed 2 W kg^−1^ for the human body and head, and 4 W kg^−1^ for the limbs [105–108].

Figure 5b shows the experimental environment consisting of the probe antenna, phantom, and dipole antenna for SAR measurement. The SAR quantifies the impact of EM fields on human exposure, representing the permissible level of radiation absorption per kilogram of human body weight [109]. The FPG was placed under the ELI Phantom V6.0. which is a solution with similar properties to human tissue to prevent the propagation of EM signals from the dipole antenna to the probe antenna. The SAR was evaluated at 5.2 GHz and 5.5 GHz, which are representative frequencies commonly utilized for indoor and outdoor wireless communication, respectively. The transmitted power was limited to 100 mW, which is the maximum power permitted for a smartphone. Before applying the FPG as shown in Fig. 5c, the measured SARs were 7.7 and 8.5 W kg^−1^ at 5.2 and 5.5 GHz, respectively. After the FPG was applied, the SAR values decreased remarkably to 1.51 and 1.89 W kg^−1^ at 5.2 and 5.5 GHz, respectively. This illustrated that FPG has notable EM wave shielding effect, resulting in significant SAR reductions of 80.3% and 77.8% at 5.2 and 5.5 GHz, respectively. Furthermore, the measured SARs at 5.2 and 5.5 GHz were both below 2 W kg^−1^, which meets the IEEE standard for exposure to the human body, head, and limbs.

To evaluate the EM wave shielding ability in wearable applications, the FPG was attached to the front of a person’s midsection, placed between the transmitter and receiver of EM waves, as shown in Fig. 5d. In practical signal communication environments, communication often occurs over long distances in the far-field, between the transmitter and receiver [110]. To simulate this in our study, we positioned the transmitter and receiver more than 3 m apart. The FPG could be conformally attached to the human body due to its flexible properties, and its lightweight allowed the person to move freely during the experiment. The transmission from the transmitter antenna to the receiver antenna was calculated as the power ratio of the transmitted and received waves, as shown in Fig. 5e. The FPG effectively shielded EM waves with frequencies ranging from 5 to 11 GHz, resulting in a reduction of the magnitude of the transmission by − 10.4 dB at 5.5 GHz. These results indicate that FPG is a strong candidate for EMI shielding materials in wearable applications, due to its lightweight, flexibility, and large size.

## Conclusion

In summary, we developed a novel high-throughput synthesis technique of flash-induced porous graphene via synergistic photo-effects for EMI shielding. Flash irradiation with a broad spectrum causes a series of photoreactions in the PI film by simultaneously absorbing UV and Vis–NIR wavelengths, leading to the synthesis of porous graphene over a large area in a few milliseconds (ms). The UV wavelengths within the full spectrum induce defects in the PI film, which enhance the absorption of Vis–NIR wavelengths and promote photothermal reactions, resulting in the synthesis of porous graphene. The XPS and FTIR results demonstrate that the carbon content becomes dominant due to the gas release accompanied by a decrease in nitrogen and oxygen content. Raman spectra, XRD and TEM images show the formation of multilayer graphene through the rearrangement of carbon atoms in a disordered carbon matrix. At a lamp fluence of 22 J cm^−2^, hollow pillar graphene was formed with a low sheet resistance of 18 Ω sq^−1^ by the conductive network as well as low density (0.0354 g cm^−3^) and a high absolute EMI SE of 1.12 × 10^5^ dB cm^2^ g^−1^ by the porous structure. The FPG retained 1.4 and 0.86 times its initial sheet resistance and SE performance, respectively, even after 10,000 bending tests. The FPG, effectively shields internal and external EMI of a drone radar system, reduces the radiated electric field (− 10.8 dB) and antenna gain (− 12.2 dB), and detects radar signals without saturation. In wearable applications, the FPG reduced SAR by 80.3% and 77.8% for indoor and outdoor frequencies, respectively, satisfying the IEEE standard. We believe that the proposed lightweight, flexible, and highly productive FPG will be effective in resolving diverse EMI shielding issues for future mobility and wearable applications.

### Supplementary Information

Below is the link to the electronic supplementary material.

Supplementary file1 (PDF 2048 kb)
