# Supplementary material for: Flash-Induced High-Throughput Porous Graphene via Synergistic Photo-Effects for Electromagnetic Interference Shielding
Source: Nanomicro Lett. 2023 Aug 2;15:191. doi: 10.1007/s40820-023-01157-8 (PMC10397175; doi:10.1007/s40820-023-01157-8)
Supplement: Supplementary file 1 — Supplementary file1 (PDF 2048 kb) [file 40820_2023_1157_MOESM1_ESM.pdf]

Supporting Information for

Flash-Induced High-Throughput Porous Graphene via Synergistic Photo-Effects for Electromagnetic Interference Shielding

Jin Soo Lee[1, #], Jeong-Wook Kim[2,#], Jae Hee Lee[1], Yong Koo Son[1], Young Bin Kim[1], Kyoohee Woo[3], Chanhee Lee[2], Il-Doo Kim[1], Jae Young Seok[4], Jong Won Yu[2], Jung Hwan Park[5, *] and Keon Jae Lee[1, *]

[1] Department of Materials Science and Engineering, Korea Advanced Institute of Science and Technology (KAIST), 291 Daehak-ro, Yuseong-gu, Daejeon 34141, Republic of Korea

[2] School of Electrical Engineering, Korea Advanced Institute of Science and Technology (KAIST) 291 Daehak-ro, Yuseong-gu, Daejeon 34141, Republic of Korea

[3] Department of Printed Electronics, Nano-Convergence Manufacturing Systems Research Division, Korea Institute of Machinery and Materials (KIMM), 156 Gajeongbuk-Ro, Yuseong-Gu, Daejeon, 34103, Republic of Korea

[4] Department of Mechanical System Design Engineering, Seoul National University of Science and Technology, 232 Gongneung-ro, Nowon-gu, Seoul, 01811, Republic of Korea

[5] Department of Mechanical Engineering (Department of Aeronautics, Mechanical and Electronic Convergence Engineering), Kumoh National Institute of Technology, 61 Daehak-ro, Gumi, Gyeongbuk, 39177, Republic of Korea

# Jin Soo Lee and Jeong-Wook Kim contributed equally to this work.

*Corresponding authors. E-mail: keonlee@kaist.ac.kr (Keon Jae Lee), parkjh1151@kumoh.ac.kr (Jung Hwan Park)

Supplementary Figures and Tables

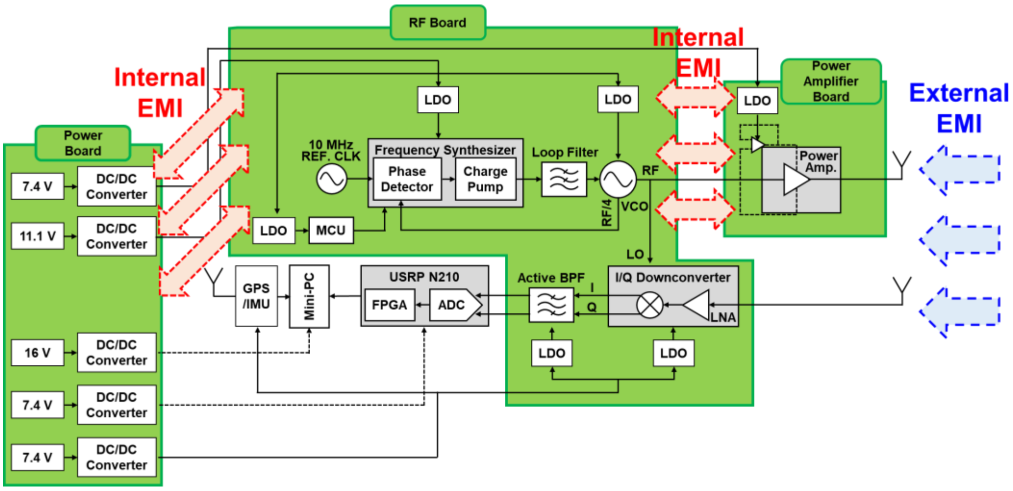

Fig. S1 Block diagram of K-band frequency-modulated continuous wave (FMCW) radar

Figure S1 shows a block diagram of K-band frequency-modulated continuous wave (FMCW) radar. The radar consists of a power board, a radio frequency (RF)

transceiver board, a power amplifier board, and antennas. The power board distributes the power required for the RF transceiver and power amplifier. The power amplifier board amplifies the transmitting signal of the radar. The RF transceiver board generates the transmitting signal and receives the reflected signal from targets. Universal software radio peripheral (USRP) N210 and Mini-PC (DFR0444, Lattepanda) were employed for capturing and saving the received radar signal.

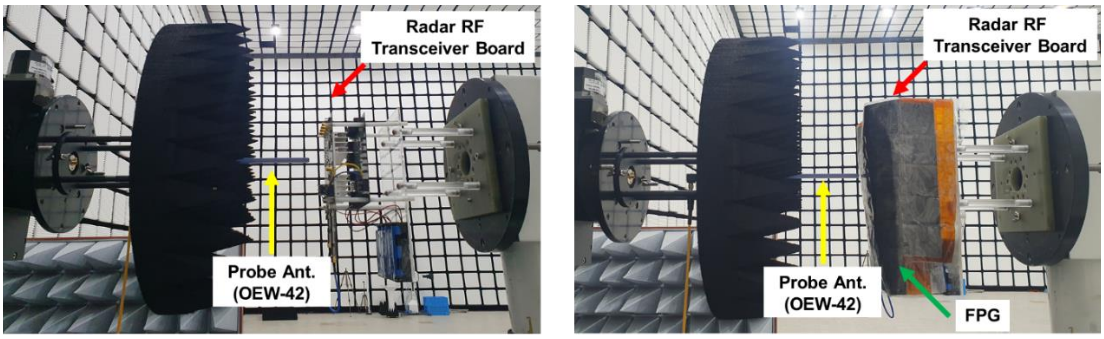

Fig. S2 Experiment environment to measure 2-dimensional electric field distribution of radar RF board without FPG and with FPG

Figure S2 shows the experiment environment to measure the 2-dimensional electric field distribution of the radar RF board with and without the FPG. By controlling the position of the probe antenna, the electric fields radiated from the RF transceiver PCB were 2-dimensionally measured in the area of 20 x 20 cm^2.

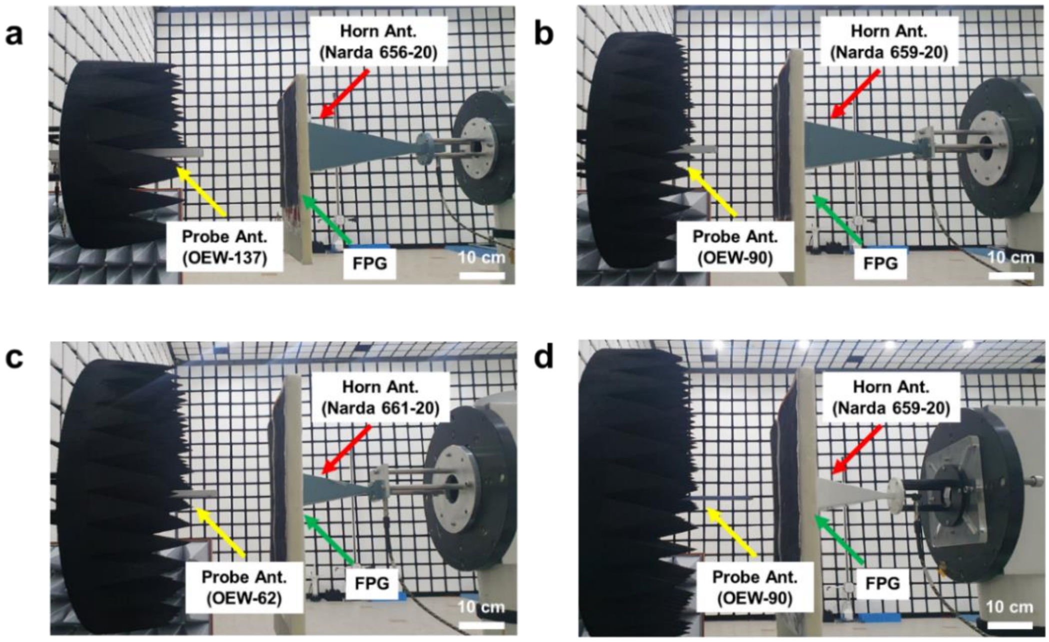

Fig. S3 a Experimental environment for measuring radiation pattern of standard Horn antenna with FPG in 5.8 ~ 8.2 GHz. **b** Experimental environment for measuring radiation pattern of standard Horn antenna with FPG in 8.2 ~ 12.4 GHz. **c** Experimental environment for measuring radiation pattern of standard Horn antenna with FPG in 12.4 ~ 18 GHz. **d** Experimental environment for measuring the radiation pattern of a standard Horn antenna with the FPG in 26.5 ~ 40 GHz

Figure S3 shows the experimental environments for measuring the radiation pattern of standard Horn antennas with various frequency bands. Various types of

standard horn antennas (Narda 656-20 for 5.8 ~ 8.2 GHz, Narda 659-20 for 8.2 ~ 12.4 GHz, Narda 661-20 for 12.4 ~ 18 GHz, and MTG SGH-28 for 26.5 ~ 40 GHz) and open-ended probe antennas (OEW-137 for 5.8 ~ 8.2 GHz, OEW-90 for 8.2 ~ 12.4 GHz, OEW-62 for 12.4 ~ 18 GHz, and OEW-28 for 26.5 ~ 40 GHz) were used.

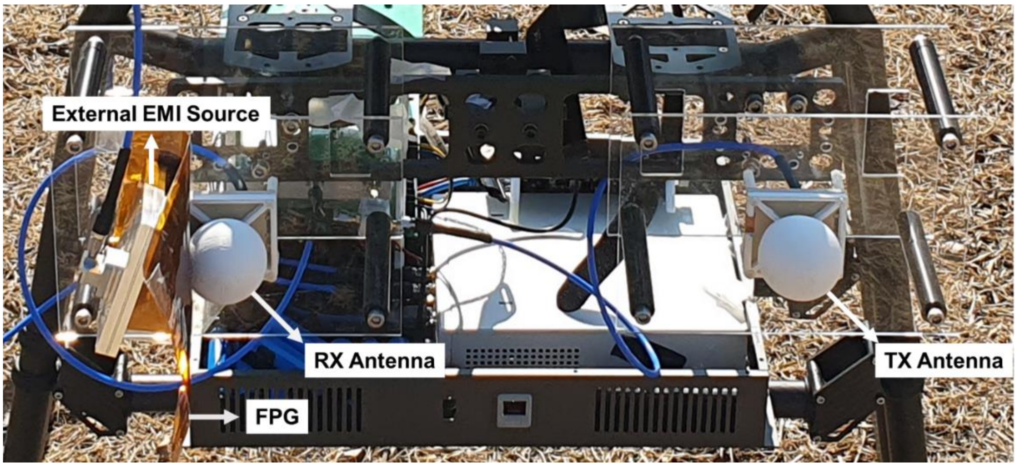

Fig. S4 Experimental environment of external EMI shielding for radar target detection

Figure S4 shows the experimental environment to evaluate external EMI shielding for radar target detection. The external EMI signal was divided from the transmitting signal of the radar. A high-gain waveguide antenna was used as an external EMI source and radiated the external EMI signal to the receiver antenna of the radar. The FPG was applied between the waveguide antenna and the receiver antenna of the radar.

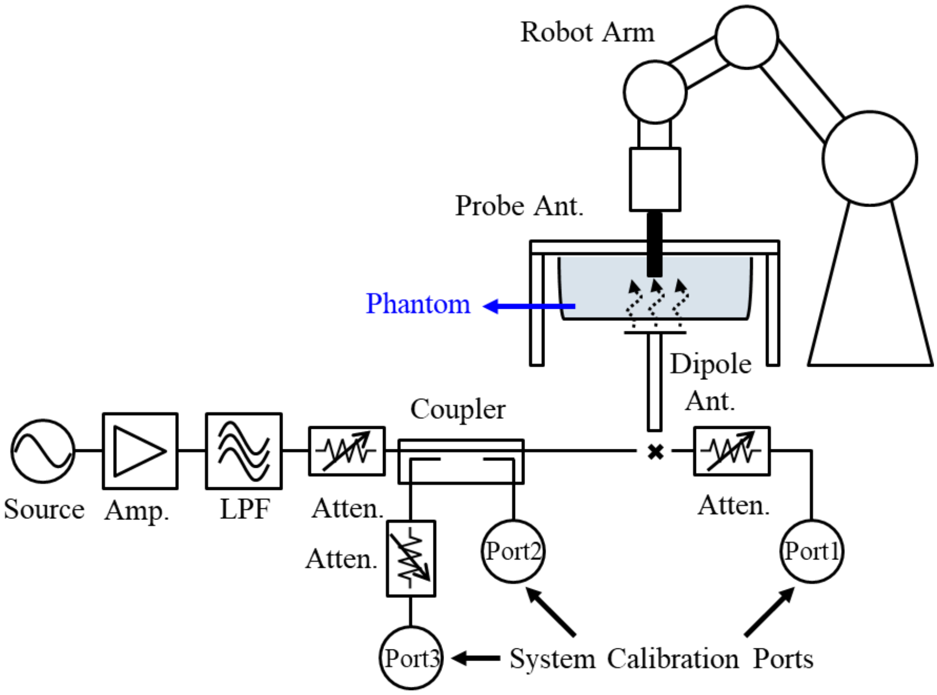

Fig. S5 Block diagram of specific absorption rate measurement system

Figure S5 shows a block diagram of the specific absorption rate measurement system. The signal source was implemented as a signal generator (N5171B) and a power amplifier (AMP2027) was used to amplify the source signal. A LPF (low pass filter, L14012FL) was used for filtering the signal source noise. A 3-dB attenuator

(8491B) was employed to control the signal power. A dual-directional coupler (772D) was used for the system calibration ports. A 10 dB attenuator was inserted for port1 and a 20 dB attenuator was adopted for port3. Using these ports, the transmitted signal was tracked in real-time for system calibration. For the transmitting antenna, a dipole antenna (D5GHZV2) was adopted, and for the receiving antenna, a probe antenna (EX3DV4) was selected. A robot arm to control the position of the probe antenna was implemented by TX90XL and for a phantom, which is a solution with similar properties to human tissue, ELI Phantom V6.0 was used. The received signal from the probe antenna was received by a power detector (U8481A).

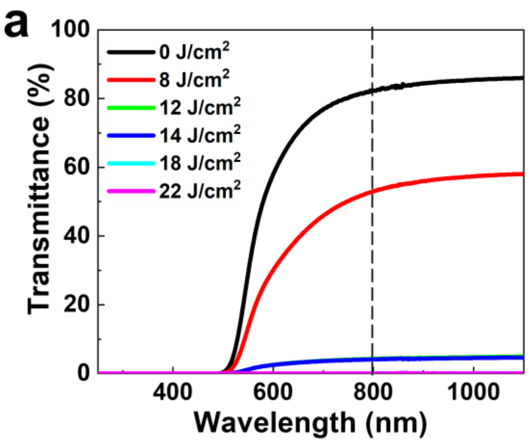

Fig. S6 Optical transmittance of PI irradiated various lamp fluences from 0 to 22 J/cm^2 using **a** Full-spectrum and **b** Vis-NIR wavelengths. The dotted line represents the transmittance at 800nm

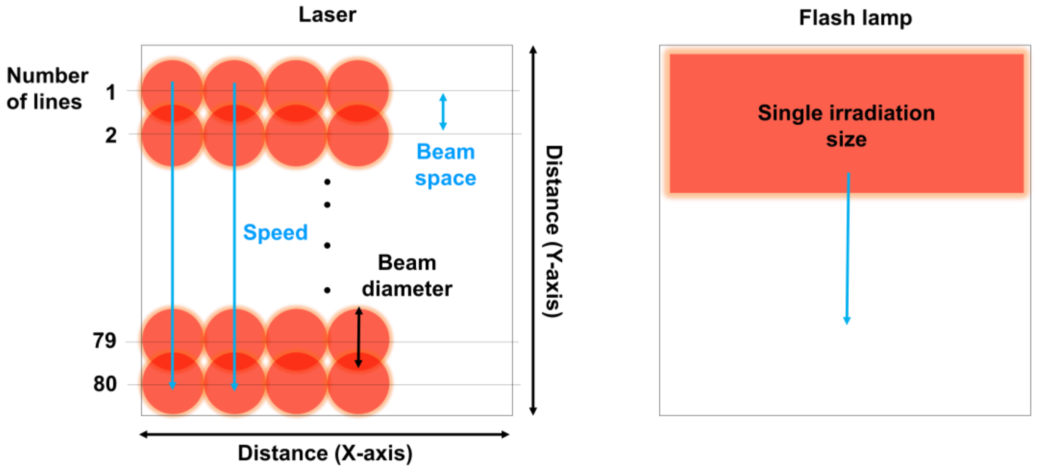

Fig. S7 Schematic of various parameters for processing time calculation

To compare processing times, the time it takes for a beam to scan the entire given area was calculated. Each parameter was referenced in the experimental section of the reference, and the calculation method is as follows (S1-S5) [S1-S3],

$$\text{Beam overlapping} = \left(1 - \frac{\text{scan speed (mm/s)}}{\text{frequency (Hz)} \times \text{beam diameter(mm)}}\right) \times 100 \quad \text{(S1)}$$

$$\text{Beam space} = \text{beam diameter} - \frac{\text{beam diameter (mm)} \times \text{beam overlapping (\%)}}{100} \quad \text{(S2)}$$

$$\text{Number of lines (\#)} = \frac{\text{Distance (Y axis,mm)}}{\text{Beam space (mm)}} \quad \text{(S3)}$$

$$\text{Scan time per line (s)} = \frac{\text{Distance (X axis,mm)}}{\text{Scan speed (mm/s)}} \quad \text{(S4)}$$

$$\text{Area process time (s)} = \text{Number of lines (\#)} \times \text{Scan time per line (s)} \quad \text{(S5)}$$

Single-pulse irradiation of the flash lamp can be achieved within milliseconds, the frequency is limited to prevent thermal damage to the equipment during continuous processing. In this experiment, the processing time was calculated and the sample was produced by fixing the frequency at 1 Hz for continuous processing.

Table S1 Comparison of processing time with calculation between this work and the previous reports using a laser

		10 × 10 mm^2			100 ×100 mm^2			200 × 200 mm^2			
Beam space (mm)	Scan speed (mm/s)	# of lines	Scan time per line (s)	Area process time (s)	# of lines	Scan time per line (s)	Area process time (s)	# of lines	Scan time per line (s)	Area process time (s)	Refs.
0.12	586	83	0.017	1.411	833	0.17	141.61	1666	0.34	566.44	[S4]
0.1	200	100	0.05	5	1000	0.5	500	2000	1	2000	[S5]
0.03	1000	333	0.1	3.33	3333	1	333.3	6666	2	1333.2	[S6]
0.05	1500	200	0.006	1.3	2000	0.066	133.3	4000	0.133	533.3	[S7]
0.25	150	40	0.06	2.4	400	0.6	240	800	1.2	960	[S8]
50 x100	100	# of Shot 1	4ms	4ms	# of Shot 2	1 at 1Hz	2	# of Shot 8	1 at 1Hz	8	FPG

Table S2 The comparison of the light-induced porous graphene from this work with the previously reported method

	Laser induced photo-reduction	Lamp induced photo-reduction	Laser induced Photo-pyrolysis	This work
Carbon precursor	Graphene oxide (GO)	Graphene oxide (GO)	PI	PI
Light source	Laser	Lamp	Laser	Lamp
Light wavelength	Mono-spectrum	Broad-spectrum	Mono-spectrum	Broad-spectrum (Synergistic-effect)
Beam profile	Gaussian	Flat-top	Gaussian	Flat-top
Large area process compatibility	x	o	x	o
Beam size	μm ~ mm	mm ~ cm	μm ~ mm	mm ~ cm
References	[S9]	[S10]	[S4]	-

Table S3 Comparison of the analysis results of the FPG with those of previous studies on light-induced graphene

Ref	[S4]	[S11]	[S12]	[S13]	[S14]	This Work
Carbon precursor	PI film	PI + urea	PI film	Poly(Ph-ddm) Film	PGE-a	PI Film
Light source	Laser	Laser	Laser	Laser	Laser	Flash lamp
Produced material	graphene					
Produced material thickness	~ 25 µm	~ 15 µm	-	~ 50 µm	~50 µm	~ 62 µm
Raman spectroscopy, observed Peak	D,G and 2D	D,G and 2D	D,G and 2D	D,G and 2D	D,G and 2D	D,G and 2D
Raman, I_D/I_G ratio	< 1	> 1	<1	~ 0.65	~ 0.8	~ 0.8
Raman, I_{2D}/I_G ratio	-	~0.4	-	-	~ 0.3	~0.38
XPS, atomic percent	C 1s > 97 %	C 1s > 90 %	-	C 1s > 92 %	C 1s > 97 %	C 1s > 96 %
XRD, peak(2θ)	25.9° / 42.9°	23.65° / 42°	25.8°	25.8° / 43°	26° / 42°	26° / 43°
TEM, interlayer distance	~3.37 Å	~3.8 Å	~3.4 Å	~3.5 Å	~3.42 Å	~3.3Å

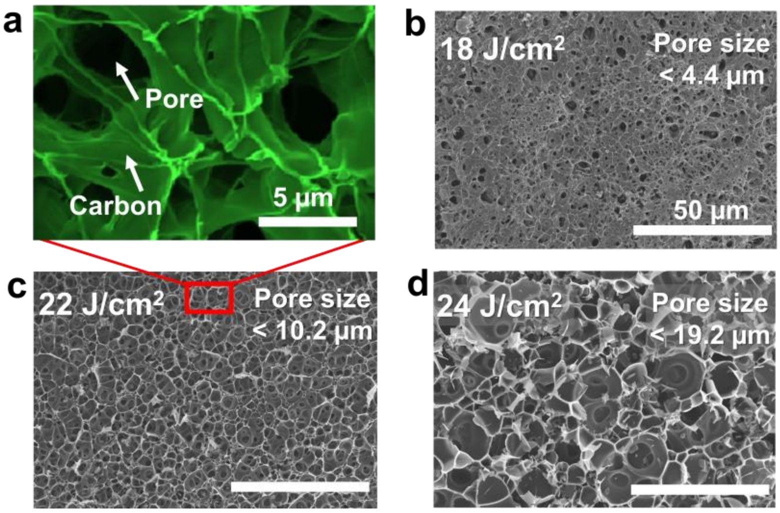

Fig. S8 **a** SEM-EDS mapping magnified image at lamp fluence of 22 J/cm^2. Green color represents carbon element. Surface SEM images of the FPG under various fluences: **b** 18, **c** 22 and **d** 24 J/cm^2. The pore size according to the lamp fluence is approximately 1.4 ~ 4.4 µm at 18 J/cm^2, 5.1 ~ 10.2 µm at 22 J/cm^2, and 7.8 ~ 19.2 µm at 24 J/cm^2, respectively

To additionally characterize the structure of synthesized materials, we performed an EDS mapping, as shown in Fig. S8a. The carbon element constituting graphene was expressed as green color and distinguished pores were verified in the black area. In addition, we confirmed flash lamp effects on pore formation by irradiating various lamp fluence from 18 to 24 J/cm^2 to the PI film. As shown in Fig. S8b, c and d, the pore size was enlarged from ~1.4 μm to ~19.2 μm in accordance with increasing lamp energy due to the difference in the quantity and emission speed of gas generated from PI [S15].

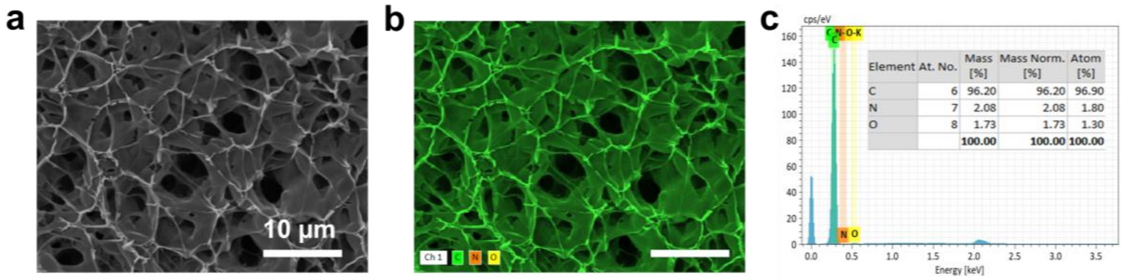

Fig. S9 a surface SEM image, **b** Element mapping image and **c** Mass (%) data by SEM-EDS of the FPG at a fluence of 22 J/cm^2

Figure S9 presents the results of elemental mapping of the FPG using SEM-EDS (Energy Dispersive X-ray Spectroscopy). Fig. S9a shows the surface SEM image of the FPG. Fig. S9b displays an image of the elements carbon, nitrogen, and oxygen, represented by green, orange, and yellow, respectively. As demonstrated in Fig. S9c, the mass percentage of carbon appears to be 96.2%. The results of the elemental mapping analysis indicate that nitrogen and oxygen from the PI are decomposed, leaving a dominant presence of carbon.

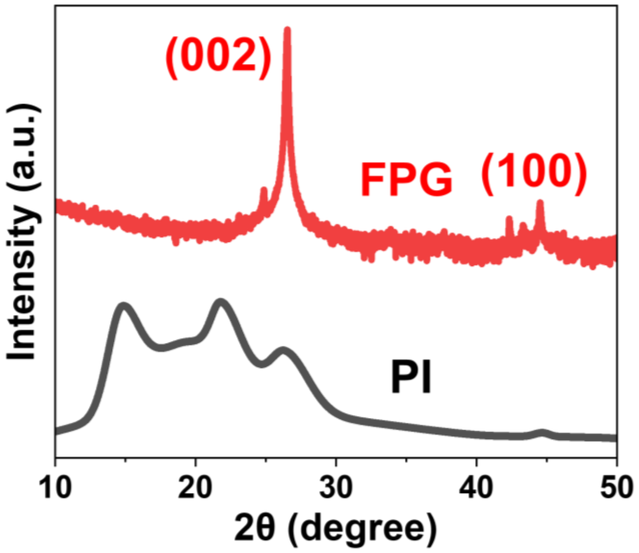

Fig. S10 X-ray diffraction (XRD) spectra of the FPG obtained at a fluence of pristine and 22 J/cm^2

The crystallinity of the material can be confirmed through XRD peaks. Fig. S10 shows an intense peak centered at $2\theta = 26°$, giving an interlayer spacing (d) of ~3.4 Å between (002) planes in the FPG, indicating a high degree of graphitization. The interlayer spacing calculated through the XRD peak is derived using the following formula:

$$n\lambda = 2d\sin\theta$$

where n represents the order of diffraction, λ is the wavelength, d is the interlayer spacing between corresponding points in adjacent crystal planes and θ is the Bragg angle. This result is consistent with measurements obtained using TEM. The peak at 2θ = 43° is indexed to (100) reflections which are associated with an in-plane structure. These indicate the formation of crystalline graphene in the FPG.

In XRD analysis, a notable distinction between graphite oxide and graphene is that the (002) peak of graphene oxide appears slightly lower and broader. Furthermore, the existence of a characteristic diffraction peak at 2θ = 11.4°, corresponding to the (001) plane, affirms the oxidation of the graphitic structure and the formation of oxygenated functional groups such as carbonyl, carboxyl, epoxide, and hydroxyl groups in graphite oxide [S16]. Amorphous carbon, on the other hand, exhibits a broad peak within 2θ = 20 ~ 30° on the XRD spectrum [S17].

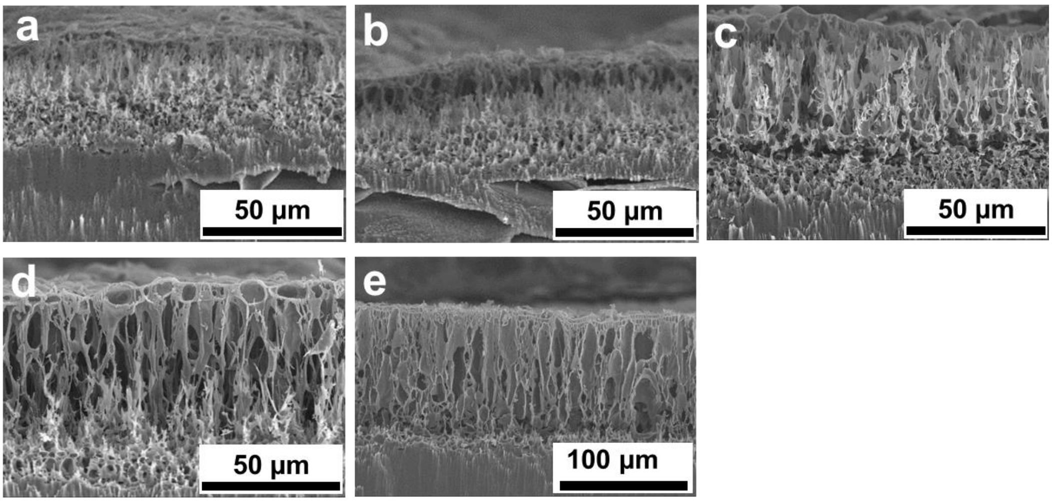

Fig. S11 Cross-sectional SEM images of the FPG under various fluences: **a** 12, **b** 14, **c** 18, **d** 22, and **e** 24 J/cm^2

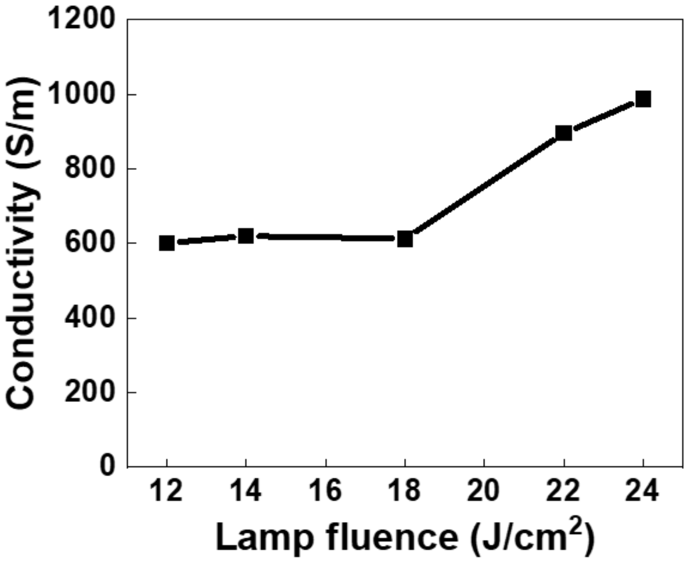

Fig. S12 The conductivity of the FPG at various lamp fluences of 12, 14, 18, 22, and 24 J/cm^2

The electrical conductivity of the FPG was calculated using the given equation and the conductivity values were plotted as a function of lamp fluence in Fig. S12.

$$\text{Conductivity (S/m)} = \frac{1}{\text{Sheet resistance}(\Omega/\text{sq}) \times \text{Thickness (m)}}$$

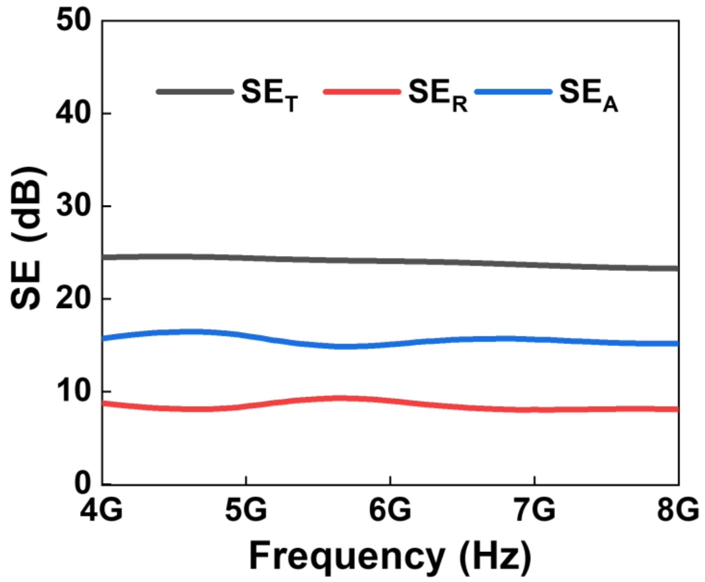

Fig. S13 EMI SE at C-band (4 ~ 8 GHz). SE_R, SE_A, and SE_T are reflection, absorption, and total EMI SE, respectively. Shielding performance exceeds 20 dB in the C-band, a value applicable in practical applications

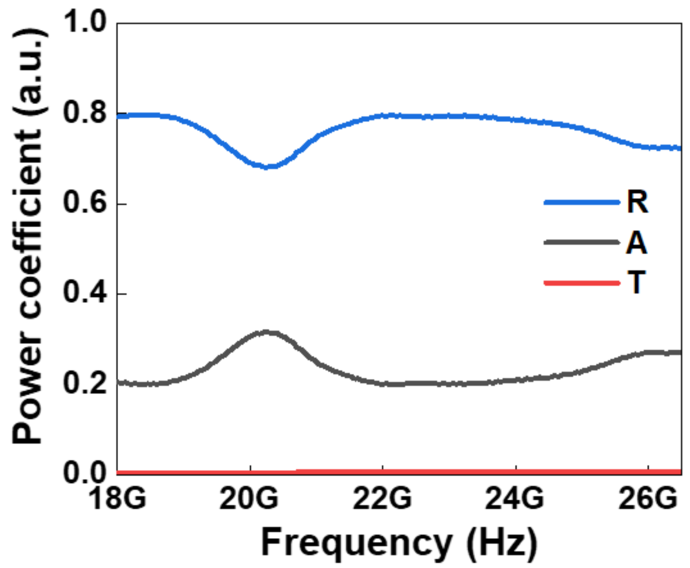

Fig. S14 R, A and T coefficients under various frequencies at a fluence of 22 J/cm^2. R, T, and A each represent reflection, transmission, and absorption, respectively

Figure S14 shows the power coefficients at K-band. The power coefficients are used to explain the mechanism of EMI shielding. R, T, and A each represent reflection, transmission, and absorption, respectively. As depicted in Fig. S14, the R value is higher than A across all frequencies, indicating that the material exhibits EMI shielding characteristics predominantly through reflection.

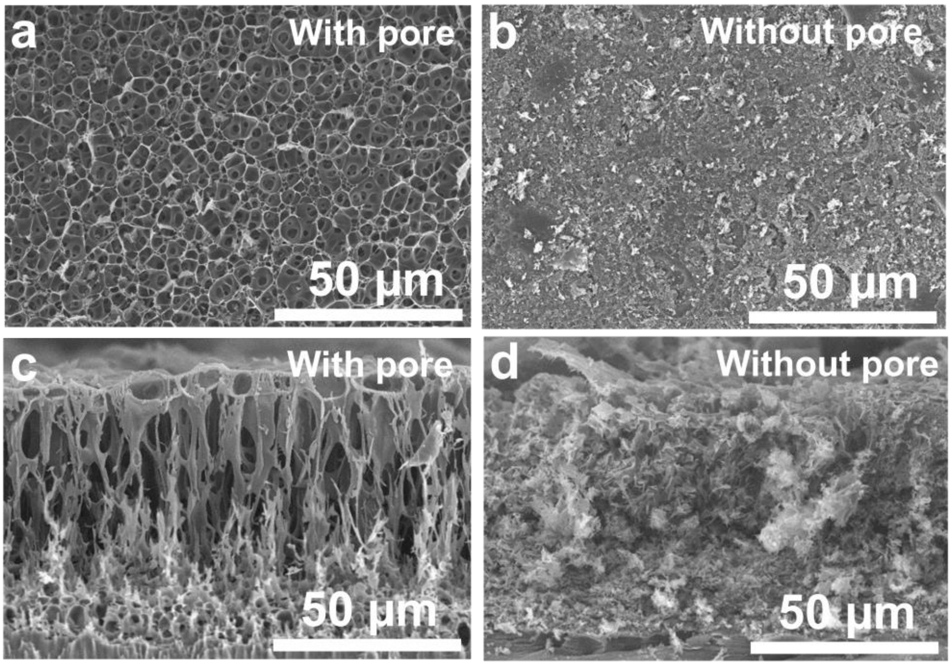

Fig. S15 SEM images of the FPG without micro-pores. Surface images **a** with pores, **b** without pores. Cross-sectional images **c** with pores, **d** without pores. The FPG formed without micro-pores has unaligned structure due to insufficient time for pore expansion during the manufacturing process

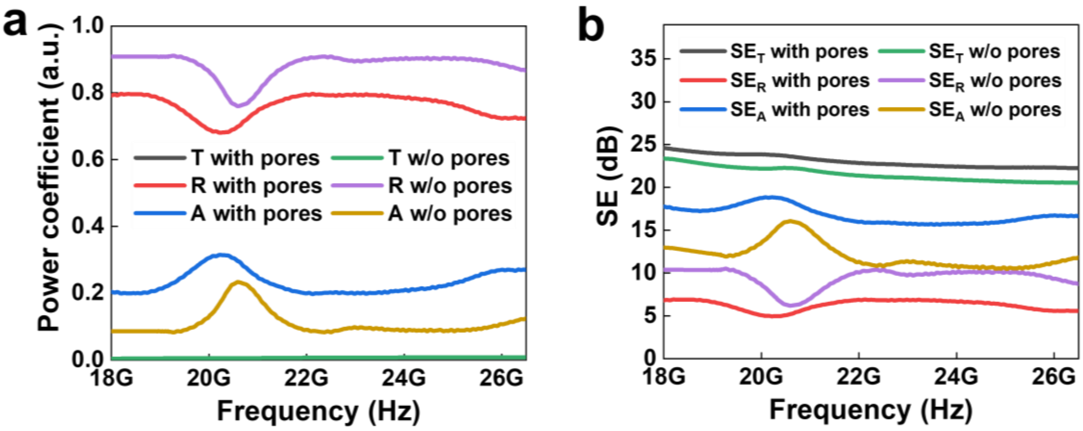

Fig. S16 a T, R and A coefficients of flash induced graphene with pores and without pores. T, R, and A represent the transmission, reflection, and absorption coefficients, respectively. The sample with micro-pores had a higher A coefficient, and a lower R coefficient than that without micro-pores. **b** EMI SE_T of flash induced graphene with pores and without pores at K-band. Although the SE_T values are similar, for the graphene with pores, the SE_A value is higher and the SE_R value is lower

The porous structure is known to affect internal scattering due to an increase in internal surface area, which is related to EM wave absorption [S18]. To verify the EMI shielding characteristics solely due to porosity, we compared power coefficient and SE between the samples with and without micro-pores at a similar thickness (Fig. S15). As shown in Fig. S16a, the sample with micro-pores had a higher A coefficient, and a lower R coefficient than that without micro-pores. The sample with micro-pores also showed a higher SE_A value (as shown in Fig. S16b) although SE_T values of the two samples

were similar. These results indicate that the porous structure enhanced EMI shielding properties by increasing EM wave absorption.

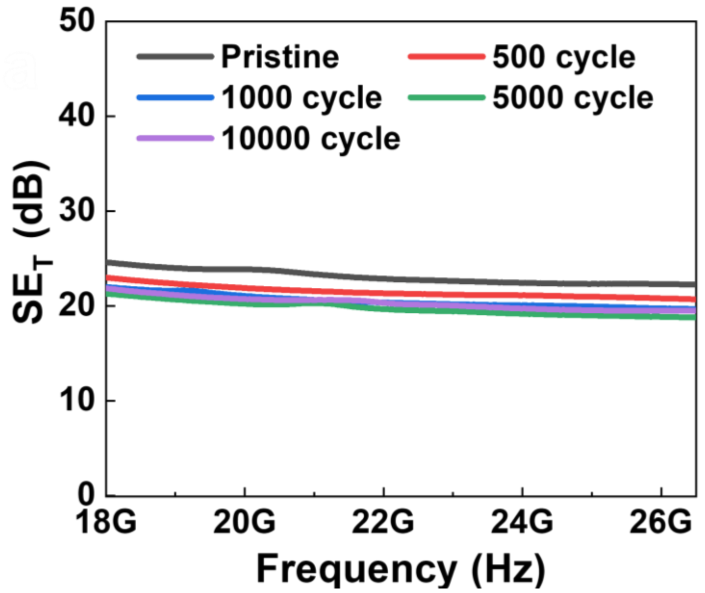

Fig. S17 SE_T results at pristine, 500, 1000, 5000, and 10000 cycles

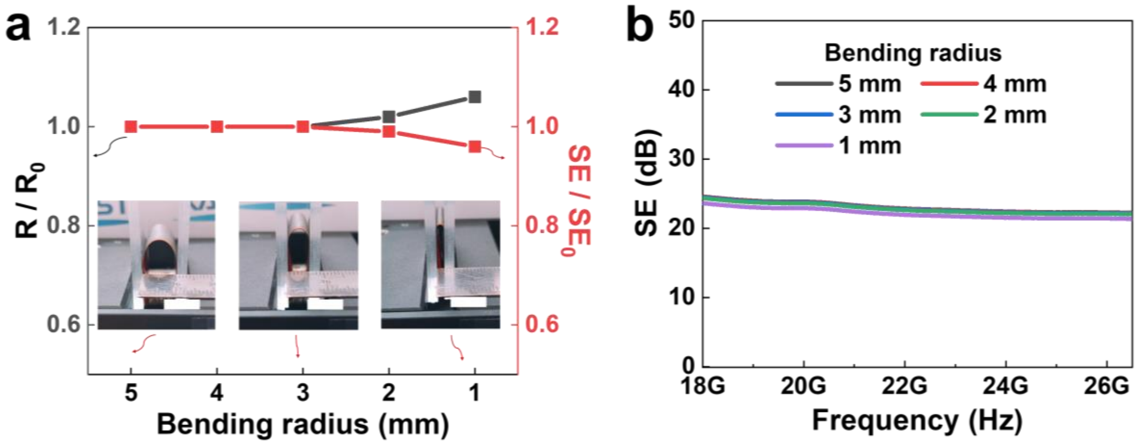

Fig. S18 a Changes in sheet resistance and EMI SE after bending with various bending radii ranging from 5 mm to 1 mm. The inset is an optical image (scale bar: 10 mm) of the FPG in a bent state. **b** EMI SE at the K-band after bending with various bending radii from 5 mm to 1 mm. From a bending radius of 5 mm down to 1 mm, the EMI SE decreased due to the formation of cracks when the bending radius was less than 3 mm

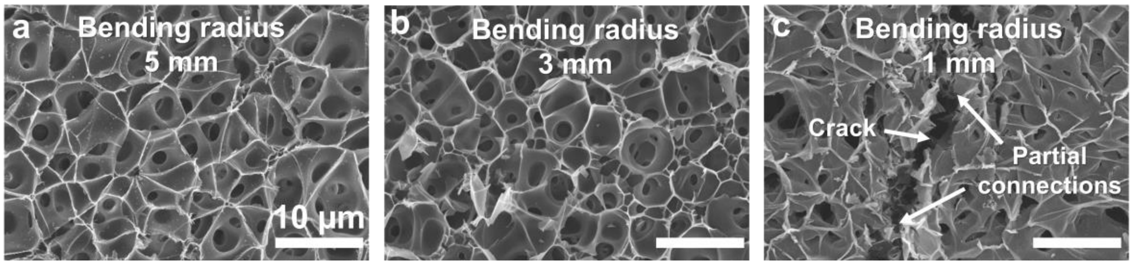

Fig. S19 SEM surface images after bending with bending radii of **a** 5 mm, **b** 3 mm, and **c** 1 mm, respectively. Cracks occur at a bending radius of less than 3mm

Table S4 Comparison of SE, SE/t, SSE and SSE/t of this work with previously reported various shielding materials

	Thickness (um)	Density (g/cm^3)	SE (dB)	SE/t (dB/cm)	SSE ($dBcm^3/g$)	SSE/t ($dBcm^2/g$)	Refs.
Carbon	300	0.28	44.5	1483.33	158.92	5297.619	[S19]
Carbon	5000	0.014	21	42	1500	3000	[S20]
Carbon	4000	0.055	40	100	727.27	1818.182	[S21]
CNT	130	0.846	65	5000	76.83	5910.165	[S22]
CNT	1800	0.01	54.8	304.44	5480	30444.44	[S23]
CNT	3000	0.042	104	346.66	2476.19	8253.968	[S24]
CNT	2000	0.0321	41.1	205.5	1280.37	6401.869	[S25]
CNT	2000	0.0625	71.4	357	1142.4	5712	[S26]
Graphene	2000	0.017	60.4	302	3552.94	17764.71	[S27]
Graphene	2000	0.116	30.5	152.5	262.93	1314.655	[S28]
Graphene	80	1.85	77.2	9650	41.72	5216.216	[S29]
Graphene	120	0.41	105	8750	256.09	21341.46	[S30]
Graphene	30	0.093	17	5666.66	182.79	60931.9	[S31]
Graphene	1600	0.022857	40	250	1750.01	10937.57	[S32]
Graphene	200	0.075	43.82	2191	584.26	29213.33	[S33]
Graphene	160	0.0249	18	1125	722.89	45180.72	[S34]
Graphene	4	1.49	38.1	95250	25.57	63926.17	[S35]
Graphene	200	0.078	63	3150	807.69	40384.62	[S36]
Graphene	53	0.22	32.7	6169.81	148.63	28044.6	[S37]
Graphene	2.8	2.14	39	139285.7	18.22	65086.78	[S38]
Graphene	3000	0.00578	33.3	111	5761.24	19204.15	[S39]
Graphene	5000	0.0167	63.6	127.2	3808.38	7616.766	[S40]
Graphene	2040	0.228	69.8	342.15	306.14	1500.688	[S41]
Mxene	0.04	2.39	17	4250000	7.11	1778243	[S42]
Mxene	0.94	4.3	46	489361.7	10.69	113805	[S43]
Mxene	0.17	3.29	3	176470.6	0.91	53638.48	[S44]
Mxene	500	0.041	43.7	874	1065.85	21317.07	[S45]
Mxene	3000	0.42	104	346.66	247.61	825.3968	[S24]
Metal	1.2	1.6	45	375000	28.12	234375	[S46]
Metal	8	2.7	66	82500	24.44	30555.56	[S47]
Metal	10	8.96	70	70000	7.81	7812.5	[S47]
Metal	2100	1	26.8	127.61	26.8	127.619	[S48]
Metal	5000	0.032	84	168	2625	5250	[S49]
This Work ($24J/cm^2$)	92	0.0402	34	3695.65	845.77	91931.65	
This Work ($22J/cm^2$)	62	0.0354	24.6	3967.74	694.91	112083.1	
This Work ($18J/cm^2$)	48	0.0354	18.7	3895.83	528.24	110051.8	

The EMI SE, SE/t, SSE and SSE/t were calculated using the given equations,

$$\mathrm{SE/t\,(dB/cm) = SE(dB)/Thickness\,(cm)}$$

$$\mathrm{SSE\,(dB \cdot cm^3/g) = SE(dB)/Density\,(g/cm^3)}$$

$$\mathrm{SSE/t\,(dB \cdot cm^2/g) = SE(dB)/Density\,(g/cm^3)/Thickness\,(cm)}$$

where SE is the shielding effectiveness, t is the thickness, SE/t is the absolute shielding effectiveness, SSE is the specific shielding effectiveness and SSE/t is the absolute specific shielding effectiveness.

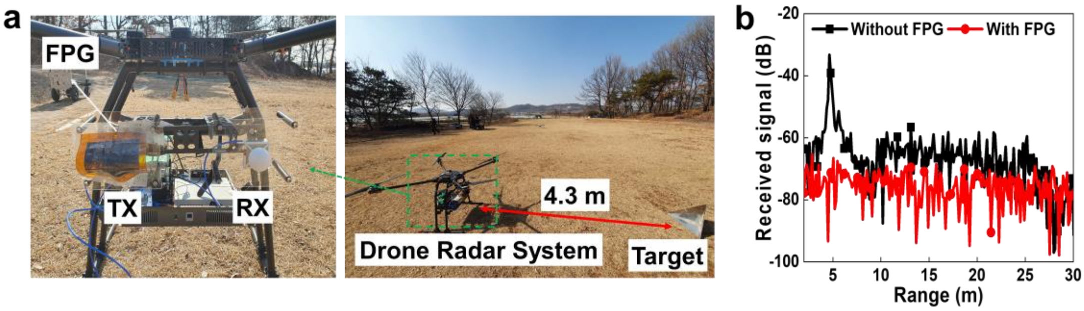

Fig. S20 a EMI shielding experiment environment for the drone radar which shows a distance of 4.3 m from the target to the FPG-applied drone radar **b** Result of the radar signal received by drone radar without and with the FPG which shows the FPG blocks the transmitter signal of drone radar well

Experiments were performed utilizing a radar system installed in a drone, as depicted in Fig. S20a. The experiment involved detecting a target located 4.3 m away using a 24 GHz radar system integrated into the vehicle. As shown in Fig. S20b, the target could be detected prior to applying the FPG. However, once the FPG was applied, the target became undetectable. These results validate the potential use of the FPG in experimental environments similar to those described in [S50].

Supplementary References

[S1] H. Palneedi, J.H. Park, D. Maurya, M. Peddigari, G.T. Hwang et al., Laser irradiation of metal oxide films and nanostructures: Applications and advances. Adv. Mater. **30**(14), 1705148 (2018). https://doi.org/10.1002/adma.201705148

[S2] G. Schnell, U. Duenow, H. Seitz, Effect of laser pulse overlap and scanning line overlap on femtosecond laser-structured ti6al4v surfaces. Mater. **13**(4), 969 (2020). https://doi.org/10.3390/ma13040969

[S3] M. Mezera, S. Alamri, W.A. Hendriks, A. Hertwig, A.M. Elert et al., Hierarchical micro-/nano-structures on polycarbonate via uv pulsed laser processing. Nanomater. **10**(6), 1184 (2020). https://doi.org/10.3390/nano10061184

[S4] J. Lin, Z. Peng, Y. Liu, F. Ruiz-Zepeda, R. Ye et al., Laser-induced porous graphene films from commercial polymers. Nat. Commun. **5**(1), 5714 (2014). https://doi.org/10.1038/ncomms6714

[S5] J. Yin, J. Zhang, S. Zhang, C. Liu, X. Yu et al., Flexible 3d porous graphene film decorated with nickel nanoparticles for absorption-dominated

electromagnetic interference shielding. Chem. Eng. J. **421**, 129763 (2021). https://doi.org/10.1016/j.cej.2021.129763

[S6] L. Huang, S. Xu, Z. Wang, K. Xue, J. Su et al., Self-reporting and photothermally enhanced rapid bacterial killing on a laser-induced graphene mask. ACS nano **14**(9), 12045-12053 (2020). https://doi.org/10.1021/acsnano.0c05330

[S7] J. Ren, D. Li, Y. Zhang, W. Yang, H.-y. Nie et al., Laser direct activation of polyimide for selective electroless plating of flexible conductive patterns. ACS Appl. Electron. Mater. **4**(5), 2191-2202 (2022). https://doi.org/10.1021/acsaelm.1c01193

[S8] D.X. Luong, K. Yang, J. Yoon, S.P. Singh, T. Wang et al., Laser-induced graphene composites as multifunctional surfaces. ACS nano **13**(2), 2579-2586 (2019). https://doi.org/10.1021/acsnano.8b09626

[S9] W. Gao, N. Singh, L. Song, Z. Liu, A.L.M. Reddy et al., Direct laser writing of micro-supercapacitors on hydrated graphite oxide films. Nat. Nanotechnol. **6**, 496-500 (2011). https://doi.org/10.1038/nnano.2011.110

[S10] L.J. Cote, R. Cruz-Silva, J. Huang, Flash reduction and patterning of graphite oxide and its polymer composite. J. Am. Chem. Soc. **131**, 11027-32 (2009). https://doi.org/10.1021/ja902348k

[S11] F. Zhang, E. Alhajji, Y. Lei, N. Kurra, H.N. Alshareef, Highly doped 3d graphene na-ion battery anode by laser scribing polyimide films in nitrogen ambient. Adv. Energy Mater. **8**, 1800353 (2018). https://doi.org/10.1002/aenm.201800353

[S12] L. Huang, M. Gu, Z. Wang, T.W. Tang, Z. Zhu et al, Highly efficient and rapid inactivation of coronavirus on non-metal hydrophobic laser-induced graphene in mild conditions. Adv. Funct. Mater. **31**, 2101195 (2021). https://doi.org/10.1002/adfm.202101195

[S13] Y. Peng, W. Zhao, F. Ni, W. Yu, X. Liu, Forest-like laser-induced graphene film with ultrahigh solar energy utilization efficiency. ACS nano **15**, 19490-502 (2021). https://doi.org/10.1021/acsnano.1c06277

[S14] W. Yu, W. Zhao, S. Wang, Q. Chen, X. Liu, Direct conversion of liquid organic precursor into 3d laser-induced graphene materials. Adv. Mater. **35**, 2209545 (2023). https://doi.org/10.1002/adma.202209545

[S15] M. Abdulhafez, G.N. Tomaraei, M. Bedewy, Fluence-dependent morphological transitions in laser-induced graphene electrodes on polyimide substrates for flexible devices. ACS Appl. Nano Mater. **4**, 2973-86 (2021). https://doi.org/10.1021/acsanm.1c00101

[S16] B. Kartick, S. Srivastava, Green synthesis of graphene. J. Nanosci. Nanotechnol. **13**, 4320-24 (2013). https://doi.org/10.1166/jnn.2013.7461

[S17] Y. Man, R.-Y. Lin, Well-dispersed double carbon layers coated on Si nanoparticles and the enhanced electrochemical performance for lithium ion batteries. J. Mater. Sci. Mater. Electron. **31**, 14912-20 (2020). https://doi.org/10.1007/s10854-020-04052-9

[S18] B. Zhao, M. Hamidinejad, S. Wang, P. Bai, R. Che et al., Advances in electromagnetic shielding properties of composite foams. J. Mater. Chem. A **9**, 8896-949 (2021). https://doi.org/10.1039/d1ta00417d

[S19] Z. Chen, D. Yi, B. Shen, L. Zhang, X. Ma et al., Semi-transparent biomass-derived macroscopic carbon grids for efficient and tunable electromagnetic shielding. Carbon **139**, 271-278 (2018). https://doi.org/10.1016/j.carbon.2018.06.070

[S20] O. Pitkänen, J. Tolvanen, I. Szenti, A.k. Kukovecz, J. Hannu et al., Lightweight hierarchical carbon nanocomposites with highly efficient and tunable electromagnetic interference shielding properties. ACS Appl. Mater. Interfaces **11**(21), 19331-19338 (2019). https://doi.org/10.1021/acsami.9b02309

[S21] L. Vazhayal, P. Wilson, K. Prabhakaran, Waste to wealth: Lightweight, mechanically strong and conductive carbon aerogels from waste tissue paper for electromagnetic shielding and co2 adsorption. Chem. Eng. J. **381**, 122628 (2020). https://doi.org/10.1016/j.cej.2019.122628

[S22] S. Lu, J. Shao, K. Ma, D. Chen, X. Wang et al., Flexible, mechanically resilient carbon nanotube composite films for high-efficiency electromagnetic interference shielding. Carbon **136**, 387-394 (2018). https://doi.org/10.1016/j.carbon.2018.04.086

[S23] D. Lu, Z. Mo, B. Liang, L. Yang, Z. He et al., Flexible, lightweight carbon nanotube sponges and composites for high-performance electromagnetic interference shielding. Carbon **133**, 457-463 (2018). https://doi.org/10.1016/j.carbon.2018.03.061

[S24] P. Sambyal, A. Iqbal, J. Hong, H. Kim, M.-K. Kim et al., Ultralight and mechanically robust ti3c2t x hybrid aerogel reinforced by carbon nanotubes for electromagnetic interference shielding. ACS Appl. Mater. Interfaces **11**(41), 38046-38054 (2019). https://doi.org/10.1021/acsami.9b12550

[S25] Y.-Y. Wang, Z.-H. Zhou, C.-G. Zhou, W.-J. Sun, J.-F. Gao et al., Lightweight and robust carbon nanotube/polyimide foam for efficient and heat-resistant electromagnetic interference shielding and microwave absorption. ACS Appl. Mater. Interfaces **12**(7), 8704-8712 (2020). https://doi.org/10.1021/acsami.9b21048

[S26] H. Liu, Y. Xu, X. Zhao, D. Han, F. Zhao et al., Lightweight leaf-structured carbon nanotubes/graphene foam and the composites with polydimethylsiloxane for electromagnetic interference shielding. Carbon **191**, 183-194 (2022). https://doi.org/10.1016/j.carbon.2022.01.051

[S27] Z. Zeng, Y. Zhang, X.Y.D. Ma, S.I.S. Shahabadi, B. Che et al., Biomass-based honeycomb-like architectures for preparation of robust carbon foams with high electromagnetic interference shielding performance. Carbon **140**, 227-236 (2018). https://doi.org/10.1016/j.carbon.2018.08.061

[S28] Y. Sun, S. Luo, H. Sun, W. Zeng, C. Ling et al., Engineering closed-cell structure in lightweight and flexible carbon foam composite for high-efficient electromagnetic interference shielding. Carbon **136**, 299-308 (2018). https://doi.org/10.1016/j.carbon.2018.04.084

[S29] F. Xu, R. Chen, Z. Lin, X. Sun, S. Wang et al., Variable densification of reduced graphene oxide foam into multifunctional high-performance graphene paper. J. Mater. Chem. C **6**(45), 12321-12328 (2018). https://doi.org/10.1039/C8TC04008G

[S30] J. Xi, Y. Li, E. Zhou, Y. Liu, W. Gao et al., Graphene aerogel films with expansion enhancement effect of high-performance electromagnetic interference shielding. Carbon **135**, 44-51 (2018). https://doi.org/10.1016/j.carbon.2018.04.041

[S31] J. Zeng, X. Ji, Y. Ma, Z. Zhang, S. Wang et al., 3D graphene fibers grown by thermal chemical vapor deposition. Adv. Mater. **30**(12), 1705380 (2018). https://doi.org/10.1002/adma.201705380

[S32] X. Zhao, L. Xu, Q. Chen, Q. Peng, M. Yang et al., Highly conductive multifunctional rgo/cnt hybrid sponge for electromagnetic wave shielding and strain sensor. Adv. Mater. Technol. **4**(9), 1900443 (2019). https://doi.org/10.1002/admt.201900443

[S33] D. Lai, X. Chen, G. Wang, X. Xu, Y. Wang, Highly conductive porous graphene film with excellent folding resilience for exceptional electromagnetic interference shielding. J. Mater. Chem. C **8**(26), 8904-8916 (2020). https://10.1039/D0TC01346C

[S34] M. Huang, C. Wang, L. Quan, T.H.-Y. Nguyen, H. Zhang et al., Cvd growth of porous graphene foam in film form. Matter **3**(2), 487-497 (2020). https://doi.org/10.1016/j.matt.2020.06.012

[S35] Q. Wei, S. Pei, X. Qian, H. Liu, Z. Liu et al., Superhigh electromagnetic interference shielding of ultrathin aligned pristine graphene nanosheets film. Adv. Mater. **32**(14), 1907411 (2020). https://doi.org/10.1002/adma.201907411

[S36] D. Lai, X. Chen, Y. Wang, Controllable fabrication of elastomeric and porous graphene films with superior foldable behavior and excellent electromagnetic interference shielding performance. Carbon **158**, 728-737 (2020). https://doi.org/10.1016/j.carbon.2019.11.047

[S37] W. Yu, Y. Peng, L. Cao, W. Zhao, X. Liu, Free-standing laser-induced graphene films for high-performance electromagnetic interference shielding. Carbon **183**, 600-611 (2021). https://doi.org/10.1016/j.carbon.2021.07.055

[S38] S. Wan, Y. Chen, S. Fang, S. Wang, Z. Xu et al., High-strength scalable graphene sheets by freezing stretch-induced alignment. Nat. Mater. **20**(5), 624-631 (2021). https://doi.org/10.1038/s41563-020-00892-2

[S39] M. Li, F. Han, S. Jiang, M. Zhang, Q. Xu et al., Lightweight cellulose nanofibril/reduced graphene oxide aerogels with unidirectional pores for efficient electromagnetic interference shielding. Adv. Mater. Interfaces **8**(24), 2101437 (2021). https://doi.org/10.1002/admi.202101437

[S40] Y. Shen, Z. Lin, J. Wei, Y. Xu, Y. Wan et al., Facile synthesis of ultra-lightweight silver/reduced graphene oxide (RGO) coated carbonized-melamine foams with high electromagnetic interference shielding effectiveness and high absorption coefficient. Carbon **186**, 9-18 (2022). https://doi.org/10.1016/j.carbon.2021.09.068

[S41] J. Xu, H. Chang, B. Zhao, R. Li, T. Cui et al., Highly stretchable and conformal electromagnetic interference shielding armor with strain sensing ability. Chem. Eng. J. **431**, 133908 (2022). https://doi.org/10.1016/j.cej.2021.133908

[S42] T. Yun, H. Kim, A. Iqbal, Y.S. Cho, G.S. Lee et al., Electromagnetic shielding of monolayer MXene assemblies. Adv. Mater. **32**, 1906769 (2020). https://doi.org/10.1002/adma.201906769

[S43] J. Zhang, N. Kong, S. Uzun, A. Levitt, S. Seyedin et al., Scalable manufacturing of free-standing, strong Ti3C2Tx MXene films with outstanding conductivity. Adv. Mater. **32**, 2001093 (2020). https://doi.org/10.1002/adma.202001093

[S44] G.M. Weng, J. Li, M. Alhabeb, C. Karpovich, H. Wang et al., Layer-by-layer assembly of cross-functional semi-transparent MXene-carbon nanotubes composite films for next-generation electromagnetic interference shielding. Adv. Funct. Mater. **28**, 1803360 (2018). https://doi.org/10.1002/adfm.201803360

[S45] Z.-H. Zeng, N. Wu, J.-J. Wei, Y.-F. Yang, T.-T. Wu et al., Porous and ultra-flexible crosslinked MXene/polyimide composites for multifunctional electromagnetic interference shielding. Nano-Micro Lett. **14**, 59 (2022). https://doi.org/10.1007/s40820-022-00800-0

[S46] Z. Zeng, F. Jiang, Y. Yue, D. Han, L. Lin et al., Flexible and ultrathin waterproof cellular membranes based on high-conjunction metal-wrapped polymer nanofibers for electromagnetic interference shielding. Adv. Mater. **32**, 1908496 (2020). https://doi.org/10.1002/adma.201908496

[S47] L. Xu, H. Lu, Y. Zhou, Z. Chi, Z. Li et al., Ultrathin, ultralight, and anisotropic ordered reduced graphene oxide fiber electromagnetic interference shielding membrane. Adv. Mater. Technol. **6**, 2100531 (2021). https://doi.org/10.1002/admt.202100531

[S48] H. Zhang, G. Zhang, Q. Gao, M. Tang, Z. Ma et al., Multifunctional microcellular PVDF/Ni-chains composite foams with enhanced electromagnetic interference shielding and superior thermal insulation performance. Chem. Eng. J. **379**, 122304 (2020). https://doi.org/10.1016/j.cej.2019.122304

[S49] C. Liang, Y. Liu, Y. Ruan, H. Qiu, P. Song et al., Multifunctional sponges with flexible motion sensing and outstanding thermal insulation for superior electromagnetic interference shielding. Compos. Part A Appl. **139**, 106143 (2020). https://doi.org/10.1016/j.compositesa.2020.106143

[S50] M.C. Vu, P.J. Park, S.-R. Bae, S.Y. Kim, Y.-M. Kang et al., Scalable ultrarobust thermoconductive nonflammable bioinspired papers of graphene nanoplatelet crosslinked aramid nanofibers for thermal management and electromagnetic shielding. J. Mater. Chem. A **9**, 8527-40 (2021). https://doi.org/10.1039/d0ta12306d
